# Ethnobotanical and Nutritional Evaluation of Understudied Wild Edible Fruits in South Africa: Bridging Indigenous Knowledge and Food Security: A Review

**DOI:** 10.3390/foods14101726

**Published:** 2025-05-13

**Authors:** Nonhlanhla Preduence Lubisi, Maropeng Erica Matlala, Luambo Jeffrey Ramarumo, Peter Tshepiso Ndhlovu

**Affiliations:** Faculty of Agriculture and Natural Sciences, School of Biology and Environmental Sciences, University of Mpumalanga, Private Bag X11283, Mbombela 1200, South Africa220207089@ump.ac.za (M.E.M.); luambo.ramarumo@ump.ac.za (L.J.R.)

**Keywords:** plants, food diversity, nutritional security, edible plants

## Abstract

Wild edible fruits are important for ensuring food and nutritional security, especially in developing countries like South Africa. Globally, wild edible fruits are widely distributed, and they are consumed in different parts of the world; however, they are undervalued. This systematic review consolidates existing knowledge addressing the utilization of wild edible fruits, and their nutritional benefits. A total of 74 wild edible fruit species belonging to 29 families found in South Africa were documented in this study. The nutritional composition was reported only in 41 (55.4%) fruit species. The Anacardiaceae family had the most cited species (n = 11) (14.86%), followed by the Moraceae (n = 6) (8.1%), and Cucurbitaceae and Ebenaceae, of which each had five species, each contributing 5.4% to the total documented species. *Sclerocarya birrea*. (A.Rich.) Hochst., *Mimusops zeyheri* Sond., and *Strychnos spinosa* Lam. are three of the most important wild fruit species contributing to food and nutritional security. This review revealed that there is a dearth of literature studies that have substantially documented the contribution of wild edible fruits in food and nutritional security. In this regard, a study on ethnobotanical evaluation incorporating wild edible fruits used by local people could significantly provide insights and enhance our understanding of indigenous and technological knowledge that could be utilized to strengthen rural food security.

## 1. Introduction

Globally, there are many edible plant species [1]; however, only a limited number of these species are being utilized as sustenance by humans. Food and nutrition security remain a critical global challenge [2]. According to Berry, Dernini [2], food security is defined as the condition whereby all individuals have physical, economic, and social access to adequate, nutritious, and safe food consistently, fulfilling the dietary requirements and preferences for an active and healthy lifestyle. Similarly, the Food and Agriculture Organization (FAO), International Fund for Agricultural Development (IFAD) [3] define food security as a situation whereby all people always have full access to food that is sufficient, safe, nutritious, and meets their everyday dietary needs.

El Bilali et al. [4] emphasize the fact that food and nutrition security are interconnected; however, they include distinct variances [4,5]. It is worth noting that the incorporation of these wild fruits into our diets is essential in promoting food and nutritional security [6,7,8,9]. Shackleton and Shackleton [10] reiterated that wild fruits could significantly contribute to alleviating food and nutritional insecurity. Consequently, Tebkew, Gebremariam [11] argued that, although wild edible fruits reduce food insecurity and malnutrition, they could also be envisaged as an alternative income generation stream for rural and marginalized communities since they can sell some wild fruits for passive income. However, this review was not intended to address aspects of food and nutritional insecurity, but rather to evaluate the available literature associated with wild edible fruits and their contribution to food and nutritional security, aiming to inform sustainable food supply chains and improve rural food and nutritional security. Even though these wild fruits can contribute to food and nutritional security, it is crucial to consider the potential risks associated with consuming them since some contain alkaloids, saponins, oxalates, and phytates [12]. It must be noted that some of these wild edible fruits may have adverse health effects when consumed in excess. This review was associated with the following research questions: (a) What are the wild fruits found in South Africa? (b) What is the contribution of wild fruits to food and nutritional security?

## 2. Materials and Methods

This study followed the preferred reporting items for systematic review and meta-analysis protocols (PRISMA-P) [13], whereby a systematic literature search was undertaken to gather data on the wild fruits being utilized in South Africa and how they contribute to food and nutritional security. The specific phrases and keywords that were used are “wild fruits”, “food security”, “nutritional composition”, and “South Africa”. Articles, e-books, dissertations, and theses found to have incorporated these keywords were vigorously evaluated and holistically reviewed based on the abovementioned keywords. Scientific databases and search engines, including Google Scholar, Pub Med, Google, Scopus, and Science Direct, were utilized. The inclusion criteria for the contribution of wild fruits to food and nutritional security were studies in South Africa. The exclusion criteria were studies that focused on the medicinal properties of wild fruits. The scientific names of the wild fruits included in this study were verified using *The World Flora Online* (https://www.worldfloraonline.org/, accessed on 18 April 2025). Common names were confirmed through *PlantZAfrica* (https://pza.sanbi.org/, accessed on 18 April 2025), and the conservation status was further cross-checked using the South African National Biodiversity Institute (SANBI) Red List of South African Plants (https://redlist.sanbi.org/, accessed on 18 April 2025).

## 3. Results and Discussion

### 3.1. Literature Results

**Figure 1:**
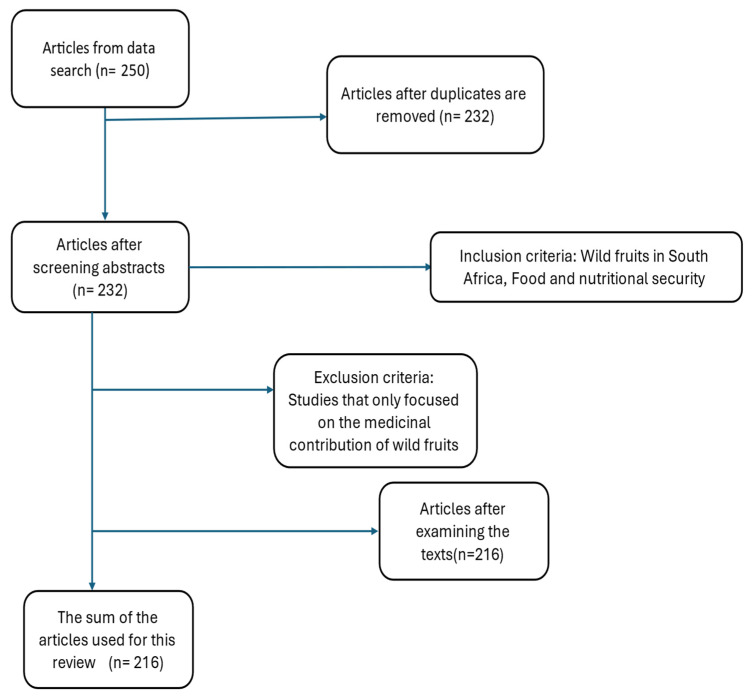
A schematic diagram showing the literature search procedure.

The literature search that was conducted using various databases initially produced 250 articles concerning wild fruits. The subsequent screening process resulted in the exclusion of 18 articles, reducing the total to 232; this screening phase is important for eliminating articles not meeting the inclusion criteria. The examination of the whole text resulted in the further exclusion of 16 more articles, yielding a final total of 216 to be considered for this review, as shown in Figure 1 above. This selection process ensures that the included studies are of relevance. Table 1 below was completed using some of the 216 articles, using the two key aspects, i.e., the contribution of wild fruits to food and nutritional security. Out of the gathered articles, a total number of 74 wild fruit species belonging to 29 different families were identified, which shows a huge diversity of wild fruits.

### 3.2. Diversity of Cited Wild Fruit Species

**Figure 2:**
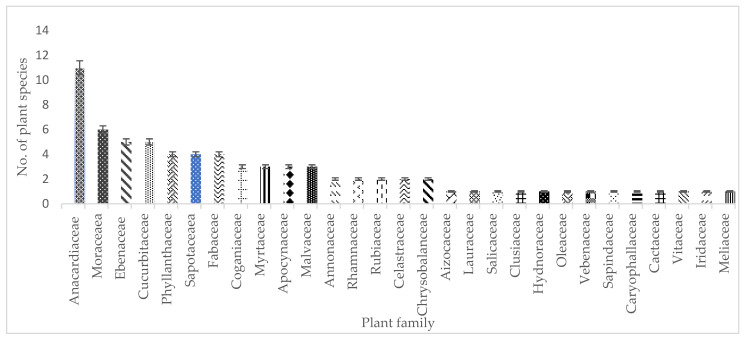
The distribution of wild fruit species per family.

In this current study, 74 wild fruit species were cited (Table 1) which belong to 29 different plant families. Anacardiaceae (n = 11) and Moraceae (n = 6) were the most dominant families, comprising 14.9% and 8.1%, respectively, followed by Cucurbitaceae (n = 5), Ebenaceae (n = 5), Phyllanthaceae (n = 4), Fabaceae (n = 4), Sapotaceae (n = 4), Loganiaceae (n = 3), Myrtaceae (n = 3), Chrysobalanceae (n = 2), Celastraceae (n = 2), Rubiaceae (n = 2), Rhamnaceae (n = 2), and Annonaceae (2), and the rest are represented by one species. The distribution of these plant species is stipulated in Figure 2 below. The results of this study align with previous studies, whereby Anacardiaceae accounted for most species [14,15,16]. Unsurprisingly, Anacardiaceae is a plant family of significant ecological and commercial importance; it is commonly known as the “cashew” family [17]. Furthermore, it encompasses significant global fruit and seed crops. According to Cunha and David [18], the plants of this family are recognized as providers of consumable fruits. The Moraceae family, that is commonly known as the “mulberry” family, includes 37 genera and roughly 1100 species that are found in tropical and temperate climates globally [19,20,21]. According to Berg [22], the fruits are often drupaceous, situated within a fleshy receptacle that constitutes a syncarp. Dejene, Agamy [23], in their study, reported that the Moraceae family has the second-highest number of wild edible fruits; these results align with the current study.

**Table 1:** The inventory of wild fruit species that are found in various parts of South Africa.

Plant Species and Family	Common Names	Distribution in South Africa	Growth Habit	Conservation Status	Food Source/Processed	Nutritional Composition	Citation
*Carpobrotus edulis* (L.) L.Bolus subsp. edulis **Aizoaceae**	Sour fig (E), vyerank (A), Wildevijg (A)	Eastern Cape, Northern Cape, and Western Cape	Herb	Least concern	Consumed raw, used to make syrup, jams, preserves, and chutney, and used as a flavor	Energy, water, protein, fat, carbohydrates, ash, Ca, Mg, Fe, Mn, Zn, Cu, Cr	[6,24,25,26,27,28]
*Harpephyllum caffrum* Bernh. **Anacardiaceae**	Wild plum (E), Umgwenya (X), Zuure Pruim (A)	Eastern Cape, KwaZulu-Natal, Limpopo, and Mpumalanga	Tree	Least concern	Consumed raw and as a snack, used to produce jams, jellies, alcoholic and non-alcoholic beverages, and rose wines	Water, protein, fat, carbohydrates, fiber, ash, Ca, Cu, Fe, Mg, Mn, Zn, Cr, vitamin A, vitamin C	[29,30,31,32,33,34,35,36]
*Lannea edulis* (Sond.) Engl. var. edulis **Anacardiaceae**	Wild Grape (E), Phepo (T), Diphiroku (P), Mutsambatsi (W), Wildedruif (A)	Free State, Gauteng, KwaZulu-Natal, Limpopo, and Mpumalanga	Shrub	Least concern	Fruit is edible, used to produce jams and jellies, consumed as a snack and sweet preserve	Fiber, carbohydrates, ash, proteins, Ca, Mg, Fe, P	[16,31,37]
*Lannea schweinfurthii* Engl. **Anacardiaceae**	Valsmaroela (A), False Marula (E), Mulivhadza (V)	KwaZulu-Natal, Limpopo, and Mpumalanga	Tree	Least concern	Fruit is edible/consumed as a snack	Not specified	[14,30,37,38]
*Ozoroa* dispar (C.Pres) R.Fern. & A.Fern. **Anacardiaceae**	Namakwa-harpuisboom (A)	Northern Cape and Western Cape	Tree	Least concern	Consumed as a snack	Not specified	[39,40]
*Sclerocarya birrea* (A.Rich.) Hochst. subsp. Caffra (Sond.) Kokwaro **Anacardiaceae**	Marula (E), Morula (TW), Cider Tree (E), Umganu (Z)	Gauteng, KwaZulu-Natal, Limpopo, Mpumalanga, and North West	Tree	Least concern	Consumed as a snack, used to make jelly, chutney, and pie fillings, nuts, used to make beer (mukumbi), fermented to make wine	Fat, water, protein, moisture, Ca, Fe, K, P, Mg, Zn, vitamins A, B_3_,C, and E, carotene	[14,15,30,41,42,43,44,45,46,47,48,49,50]
*Searsia dentata* (Thunb.) F.A.Barkley **Anacardiaceae**	Nana berry (E), Nanabessie (A)	Eastern Cape, Free State, Gauteng, KwaZulu-Natal, Limpopo, Mpumalanga, and North West	Shrub	Least concern	Used to produce milk curdles, consumed as a snack	Not specified	[31,51,52]
*Searsia discolor* (E.Mey.ex Sond.) T.S.Yi, A.J.Mill. & J.Wen **Anacardiaceae**	Grassveld Currant (E)	Eastern Cape, Free State, Gauteng, KwaZulu-Natal, Limpopo, Mpumalanga, and North West	Shrub	Least concern	Consumed as a snack	Not specified	[31,51,52]
*Searsia leptodictya* (Diels) T.S.Yi, A.J.Mill. & J.Wen **Anacardiaceae**	Rock Karee (E)	Free State, Gauteng, KwaZulu-Natal, Limpopo, Mpumalanga, and North West	Tree	Least concern	The fruit is edible, used to make yeast	Not specified	[15,31,33]
*Searsia pendulina* (Jacq.) Moffett **Anacardiaceae**	Mosilabele (S), Botlhotlho (PL), Witkaree (A)	Free State, Mpumalanga, and Northern Cape	Tree	Least concern	Fruit is edible, consumed as a snack, used to produce alcoholic beverages, eaten raw, soaked in milk, used to make porridge and milk curdles	Not specified	[14,53]
*Searsia pentheri* (Zahlbr.) Moffet **Anacardiaceae**	Mutasiri, Crow-berry (E)	Eastern Cape, KwaZulu-Natal, Limpopo, and Mpumalanga	Tree	Least concern	Consumed as a snack	Not specified	[31,33,38,52]
*Searsia undulata* (Jacq.) T.S.Yi, AJ.Mill. & J.Wen **Anacardiaceae**	Kuni-bush (E)	Northern Cape and Western Cape	Shrub	Least concern	Consumed as a snack, used to make yeast and milk curdles	Not specified	[6,39,47,53,54,55]
*Annona senegalensis* Pers. **Annonaceae**	Wild custard apple (E), isiphofu (Z), muembe (V), Wildesuikerappel (A)	KwaZulu-Natal, Limpopo, and Mpumalanga	Tree	Least concern	Consumed as fresh fruit, used to make ice-cream, sherbets, and drinks	Fe, K, P, Mg, Ca, Na, S, Cl, Al, Si, V, Cr, Mn, Fe, Ni, Cu, Zn, Se, Br, Mo, Sn, I, Ba, Pb, moisture, ash, crude fiber, crude protein, carbohydrates	[14,47,56,57,58]
*Hexalobus monopetalus* (A.Rich) Engl. & Diels **Annonaceae**	Shakama plum (E), Moheteka (NS), Custard Apple (E), Shakama-pruim (A)	Limpopo and Mpumalanga	Tree	Least concern	Consumed as a snack, used to produce sweet preserve	Not specified	[6,30,37,53]
*Ancylobotrys capensis* (Oliv.) Pichon **Apocynaceae**	Wild apricot (E), rock milk apricot (E)	Gauteng, KwaZulu-Natal, Limpopo, Mpumalanga, and North West	Shrub	Least concern	Consumed as a snack, alcoholic beverages, and savory and sweet preserves	Not specified	[6,53,59]
*Carissa macrocarpa* (Eckl.) A.D.C **Apocynaceae**	Natal plum (E), big num-num (E), nmthungulu (X), grootnoem-noem (A)	Eastern Cape and KwaZulu-Natal	Shrub	Least concern	Fruit is edible, used to produce alcoholic beverages such as wine, used as a preserve, used to produce sweets	Energy, water, proteins, fat, carbohydrates, fiber, ash, Ca, K, Mn, Zn, Fe, vitamin C	[6,15,30,46,53,60,61,62,63,64].
*Carissa spinarum* L. **Apocynaceae**	Umlugulu (ND), mothokolo (PI), ntshuguru (XT), murungulu (V)	Limpopo and Mpumalanga	Shrub	Least concern	Fruit is edible, used to create alcoholic and non-alcoholic beverages such as wine, snacks, and sweet and savory preserves	Ca, Fe, K, Zn, Mn, moisture, carbohydrates, energy, ash, fat, fiber, total protein, vitamin C	[15,46,63,65,66]
*Opuntia ficus-indica* (L.) Mill. **Cactaceae**	Bobbejaansturksvy (A), Idolofia (ND), Indian Fig (E), Kaalblad (a), Makonde (V), Spineless Cactus (E), Sweet Prickly Pear (E)	Eastern Cape and Limpopo	Shrub	Not evaluated	Consumed as a snack, used to make alcoholic beverages, savory and sweet preserves, jam, juice, and tea	Moisture content, crude fiber, crude fat, protein, sugars, carbohydrates, P, Cu, Zn, Fe, Mn, Ca, K, Na, Mg, vitamin C	[32,37,52,53,67,68]
*Pollichia campestris* Aiton **Caryophyllaceae**	Waxberry (E), umhlungulu (Z), amangabangaba (X), Suikerrteebossie (A)	Eastern Cape, Free State, Gauteng, KwaZulu-Natal, Limpopo, Mpumalanga, Northern Cape, North West, and Western Cape	Shrub	Least concern	Consumed as a snack	Not specified	[30,52,53,69]
*Mystroxylon aethiopicum* (Thunb.) Loes. subsp. Aethiopicum **Celastraceae**	Umbovane (X), Umnqayi (X), Cape cherry (E), Koeboebessie (A), Umgumguluzane (Z)	Eastern Cape and Western Cape	Tree	Least concern	Consumed as a snack	Not specified	[30,37,40,70,71]
*Salacia kraussii* Harv. **Celastraceae**	Ibonsi (Z), Ihelehele (Z), Ubangalala (Z)	KwaZulu-Natal and Limpopo	Shrub	Least concern	Consumed as a snack	Ca, Fe, K, P, Mg	[30,49,53,70,72,73]
*Parinari capensis* Harv. **Chrysobalanceae**	Gruisappeltjie (A), Dwarf Mobola-plum (E), Mmolofasane (P)	Gauteng, KwaZulu-Natal, Limpopo, and North West	Shrub	Least concern	Fruit is edible, used to produce syrup, porridge, alcoholic and non-alcoholic beverages	Proteins, moisture, amino acids	[6,14,15,30,33,37,53,74,75]
*Parinari curatellifolia* Planchon. ex Benth. **Chrysobalanceae**	Mobola Plum (E), Amabulwa (Z), Mbola (ND), Sand Apple (E), Ubulawu (Z)	Limpopo and Mpumalanga	Shrub	Least concern	Fruits are consumed in their natural state or processed into a jam, used to produce soft drinks and beer, used to make syrup	Carbohydrates, dry matter, ash, protein, fiber, fat, Ca, Fe, K, P, Mg, Mn, Cu, Zn, vitamin C	[14,15,61,76,77,78,79]
*Garcinia livingstonei* T.Anderson **Clusiaceae**	African mangosteen (E), Umphimbi (Z)	KwaZulu-Natal, Limpopo, and Mpumalanga	Tree	Least concern	Fruits are edible, used to make alcoholic beverages, consumed as a snack, milk, curdles, sweet preserve	Ca, Fe, K, P, Mg, vitamin A	[30,35,59,69,71,80,81,82]
*Acanthosicyos horridus* Welw. ex Hook.f. **Cucurbitaceae**	Nara Bush (E)	Northern Cape	Shrub	Critically endangered	Consumed as a snack, used to produce non-alcoholic and alcoholic beverages, sweet preserve, used to make milk curdles	β-carotene, ash, Ca, Cu, N, P, K, Fe, Mg, Zn, Na, crude fiber, dry matter, energy, fat, carbohydrates, nicotinic acid, protein, riboflavin, thiamine, water, vitamin C	[40,60,83,84,85,86,87]
*Citrillus lanatus* (Thunb.) Matsum. & Nakai **Cucurbitaceae**	Tsamma melon (E), Bitterappel (A), Bitter Apple (E), Wild melon (E)	Eastern Cape, Free State, Gauteng, KwaZulu-Natal, Limpopo, Mpumalanga, Northern Cape, and North West	Climber	Least concern	Eaten fresh, used to produce juice and desserts	Fe, K, P, Mg	[88,89,90,91]
*Coccinia sessilifolia* (Sond.) Cogn. **Cucurbitaceae**	Ystervarkkambroo (A), Rooi-agurkie (A)	Free State, Gauteng, Limpopo, Mpumalanga, Northern Cape, and North West	Climber	Least concern	Fruits are edible	Ca, Fe, K, P, Mg, vitamin C	[30,92]
*Cucumis metuliferus* E.Mey. Naudin **Cucurbitaceae**	Jelly melon (E), Spiny cucumber (E), Uhufafa (Z), Wildekomkommer (A)	KwaZulu-Natal, Limpopo, and Mpumalanga	Climber	Least concern	Fruits are eaten raw, can be used to make a jelly	Ca, Fe, P, Mg, Mn, Na, K, Zn, vitamin A, vitamin B_1,_ B_2,_ B_3,_ B_5,_B_6_, and B_9_, vitamin C, vitamin D, vitamin E, vitamin K	[93,94,95,96]
*Cucumis myriocarpus Naudin* subsp. leptodermis (Schweick.) C.Jeffrey & P.Halliday **Cucurbitaceae**	Paddy Melon (E), Squash melon (E), Bitter Apple (E)	Eastern Cape, Free State, KwaZulu-Natal, Northern Cape, North West, and Western Cape	Climber	Least concern	Consumed as a snack and savory preserve	Ca, Fe, K, P, Mg	[37,97]
*Diospyros lycioides* Desf. subsp. Lycioides **Ebenaceae**	Bluebush Star-apple (E), Muthala (V), Bloubos (A), Star Apple (E)	Eastern Cape, Free State, Gauteng, Limpopo, Mpumalanga, Northern Cape, North West, and Western Cape	Shrub	Least concern	Snack, used to produce alcoholic beverages	Fe, Mg, P, K, Zn, protein, water, Niacin	[30,37,40,65,69]
*Diospyros mespiliformis* Hochst. ex A.DC. **Ebenaceae**	Musuma (V), Jackal-berry (E), Hill Matome (E)	Limpopo and Mpumalanga	Tree	Least concern	Fruit is edible, eaten as a snack	Ash, protein, carbohydrates, fat, moisture, Ca, Mg, Na, K, P, S, Se, Fe, Zn, Mn, Cu	[14,15,46,98,99,100]
*Euclea crispa* (Thunb.) Gürke **Ebenaceae**	Blue guarri (E), Munyele (V), Idungamuzi (Z), Guarritee (A)	Eastern Cape, Free State, Gauteng, KwaZulu-Natal, Limpopo, Mpumalanga, North West, and Western Cape	Tree	Least concern	Consumed as a snack	Not specified	[15,30,37,52]
*Euclea divinorum* Hiern. **Ebenaceae**	Umhlangula (Z), Magic Guarri (E), Gwarriebos	KwaZulu-Natal, Limpopo, and Mpumalanga	Tree	Least concern	Fruit is eaten as a snack	Not specified	[14,30,33,37,54,69]
*Euclea natalensis* A. DC. subsp. obovata F.White **Ebenaceae**	Coast Hairy Guarri (E)	Eastern Cape and KwaZulu-Natal	Tree	Least concern	Consumed as a snack	Not specified	[7,30,37]
*Cordyla africana* Lour. **Fabaceae**	Wild mango (E), Wilde-mango (A), Umbohone (Z)	KwaZulu-Natal	Tree	Least concern	Eaten fresh or cooked	Vitamin C	[30,53,59,101]
*Dialium schlechteri* Harms **Fabaceae**	Umthiba (Z), Zulu Pod-berry (E), Zulu-peulbessie (A)	KwaZulu-Natal	Tree	Least concern	Consumed as a snack, used to produce non-alcoholic beverages	Not specified	[30,33,102]
*Macrotyloma maranguense* (Taub.) Verdc. **Fabaceae**	Mokorola kgogo (PI), Xikondlo (XT)	KwaZulu-Natal, Limpopo, and Mpumalanga	Climber	Least concern	Fruit is edible	Not specified	[14,31]
*Piliostigma thonningii (Schumach.) Milne-Redh.* **Fabaceae**	Camel’s Foot (E), Ihabahaba (ND), Kameelspoor (A), Mokgôrôpô (NS), Monkey Bread (E), Mukolokote (NS), Mukolokote (V), Picture-frame Tree (E), Rhodesian Bauhinia (E), Rhodesiese Bauhinia (A)	Limpopo and Mpumalanga	Tree	Least concern	Fruit is edible	Moisture	[15,30,33,37]
*Hydrona africana* Thumb. **Hydnoraceae**	Ubuklunga (X), Jakkalskos (A), Jackal food (E), Umavumbuka (Z)	Eastern Cape, KwaZulu-Natal, and Western Cape	Not specified	Least concern	Consumed as a snack	Ca, Fe, K, P, Mg	[6,30,103,104]
*Romulea rosea* (L.) Eckl. var. australis (Ewart) M.P.de Vos **Iridaceae**	Froetang (A), Frutang (E), Knikkertjie (A), Perdefroetang (A), Pink Romulea (E), Spruitjie (A)	Eastern Cape and Western Cape	Herb	Least concern	Consumed as a snack	Not specified	[26,38,53,55]
*Cryptocarya wyliei* Stapf **Lauraceae**	Red quince (E),UmXaleba (X), Umngcabe (Z), Rooikweper (A)	Eastern Cape and KwaZulu-Natal	Shrub	Near threatened	Consumed as a snack and sweet preserve	Not specified	[30,53]
*Strychnos cocculoides Bak.* **Loganiaceae**	Bitter Bush Orange (E), Corky Monkey-Orange (E), Grysklapper (A), Umkemeswane (ND), Umkhethswane (ND), Wynklapper (A)	Limpopo and Mpumalanga	Shrub	Least concern	Fruits are eaten raw as a snack, used to produce wine, juice and jam, used to make alcoholic beverages and porridge	Carbohydrates, protein, moisture, fat, fiber, ash, energy, Fe, P, Ca, Mg, Na, Zn, K, Cu, vitamin C	[6,30,33,37,53,54,105,106,107]
*Strychnos madagascariensis Poir.* **Loganiaceae**	Black Monkey Orange (E), Botterklapper (A), Mukwakwa (V), Swartklapper (A), Umwawa (ND),	Gauteng, KwaZulu-Natal, Limpopo, Mpumalanga, and North West	Shrub	Least concern	Fruit is edible, used to make sweets	Carbohydrates, fiber, fat, moisture, ash, protein, Na, Ca, Zn, Cu, K, Mg, N, Fe, P	[14,15,30,46,102,106,108,109,110]
*Strychnos spinosa* Lam. **Loganiaceae**	African orange (E), Umngono (ND), Wildekalabasboom (A)	Eastern Cape, KwaZulu-Natal, Limpopo, and Mpumalanga	Tree	Least concern	Fruit is edible, can produce alcoholic beverages	Carbohydrates, dry matter, proteins, moisture, fat, fiber, ash, energy Cu, Mn, K, Zn, Ca, Mg, Na, Fe, P, vitamin C	[14,15,46,77,107,111,112,113]
*Azanza garckeana* (F.Hoffm.) Exell & Hillc. **Malvaceae**	Uxaguxagu (Nd), snot apple (E), muthowa (V)	Limpopo	Shrub	Least concern	Consumed as a snack (raw)	Ca, Fe, K, P, Mg, Na, fiber, carbohydrates, ash, moisture, proteins, fat, vitamin C	[6,77,114,115,116,117]
*Grewia flava DC*. **Malvaceae**	Wild raisin (E), Velvet raisin (E), Wild currant (E), Wilderosyntjie (A)	Free State, Gauteng, KwaZulu-Natal, Limpopo, Mpumalanga, Northern Cape, and North West	Shrub	Least concern	Consumed as a snack, used to produce alcoholic beverages	Not specified	[33,53,54,106]
*Grewia flavescens* Juss. **Malvaceae**	Donkey berry (E), Ubhuzu (ND), Skurweblaarrosyntjie (A)	Gauteng, KwaZulu-Natal, Limpopo, Mpumalanga, and North West	Tree	Least concern	Fruit is edible, consumed as a snack, used to produce juice and alcohol	Carbohydrates, starch, sugar, amino acids, fats, protein, fiber, moisture, ash, Mn, Ca, K, Zn, Cu, Fe	[14,15,30,54,69,71,105,106,118]
*Trichilia dregeana* Sond. **Meliaceae**	Bos Rooi-essenhout (A), Bosrooiessenhout (A), Cape Mahogany Mmaba (NS), Mutshikili (V), Mutuhu (V), Umathunzini (Z), Umhlakele (X)	Eastern Cape, KwaZulu-Natal, Limpopo, and Mpumalanga	Tree	Least concern	Consumed as a snack	Sugar, protein, fat, moisture	[6,15,35,70,119]
*Ficus burkei* (Miq.) Miq. **Moraceae**	Common Wild Fig (E), Intenjane (ND), Moumo (NS), Umbobe (Z), Uluzi (X), Umtende (ND)	Eastern Cape, Gauteng, KwaZulu-Natal, Limpopo, Mpumalanga, and North West	Tree	Least concern	Used to produce alcoholic beverages and snacks	Not specified	[37,120]
*Ficus petersii* Warb. **Moraceae**	Wildevyeboom (A), Mmadintana (TW)	Limpopo and Mpumalanga	Tree	Least concern	Fruit is edible	Not specified	[31,121]
*Ficus sansibarica* Warb. subsp. Sansibarica **Moraceae**	Muvumo (V), Nhlampfu (TS), Zanzibar fig (E), Mudzula-tshinya (V)	Limpopo and Mpumalanga	Tree	Least concern	Snack	Not specified	[31,33,37]
*Ficus sycomorus* L. **Moraceae**	Mulberry fig (E)	KwaZulu-Natal, Limpopo, and Mpumalanga	Tree	Least concern	Coffee, snack, alcoholic beverages, sweet preserve	Dietary fiber, Ca, Fe, K, P, Mg, vitamin C	[30,59,115,122]
*Ficus sur Forssk.* **Moraceae**	Wild fig (E), bush fig (E), umkiwa (ND), Xinkuwana (TS)	Eastern Cape, KwaZulu-Natal, Limpopo, Mpumalanga, and Western Cape	Tree	Least concern	Eaten raw, used to produce fig jam or preserves	Ca, Fe, Mg, Mn	[14,30,37,65,123]
*Ficus thonningii* Blume **Moraceae**	Gewone wurgvy (A), umBombe (Z), Nhluhlawumbe (XT)	Eastern Cape, Gauteng, KwaZulu-Natal, Limpopo, Mpumalanga, and North West	Tree	Least concern	Fruit is edible, used to produce jam and alcoholic beverages	Not specified	[14,30,124]
*Syzygium cordatum* Hochst. ex C.Krauss subsp. cordatum **Myrtaceae**	Mawthoo (S), Mawtoo (S), Motlho (NS), Umjomi (X), Umswe (Z), Water Berry (E), Water Wood (E), Waterbessie (A),	Eastern Cape, KwaZulu-Natal, Limpopo, and Mpumalanga	Tree	Least concern	Fruit is edible, used to produce alcohol	Ca, Fe, K, P, Mg, vitamin C	[14,15,59,125]
*Syzygium guineense* (Willd.) DC. subsp. Guineense **Myrtaceae**	Bushveld water-berry (E), water pear (E), Mutuphemba (V)	KwaZulu-Natal, Limpopo, and Mpumalanga	Tree	Least concern	Fruit is edible	Fiber, ash, protein, fat, Ca, K, P, Ti, Mn, Fe, S, Ce, C, Zn, Al, B, Hg, Co, Cl, Zr, Pb, Mo, Sr, Zr, Ti, V, vitamin A	[30,37,46,126,127]
*Syzygium intermedium* Engl. & Brehmer **Myrtaceae**	Intermediate water berry (E)	Eastern Cape, KwaZulu-Natal, and Limpopo	Tree	Least concern	Fruit is edible, raw or as a snack	-Not specified	[14]
*Olea capensis* L. subsp. Macrocarpa (C.H. Wright) I. Verd **Oleaceae**	Ironwood (E)	Eastern Cape, KwaZulu-Natal, Limpopo, Mpumalanga, and Western Cape	Tree	Least concern	Consumed as a snack	Not specified	[33,128]
*Antidesma venosum E.Mey. ex Tul.* **Phyllanthaceae**	Segagama (T), isiqutwane (Z), umtyongi (X), Tasselberry (E)	Eastern Cape, KwaZulu-Natal, Limpopo, and Mpumalanga	Tree	Least concern	Fruit is edible, consumed as a snack	Moisture, amino acid contents, fats, sugars, protein, vitamin B_1,_ vitamin B_2,_ vitamin C, vitamin E	[15,33,35,40,46,65,75]
*Bridelia micrantha (Hochst.) Baill.* **Phyllanthaceae**	Umshonge (Z), umhlahlangu (X), motsere (NS), wild coffee (E)	Eastern Cape, KwaZulu-Natal, Limpopo, and Mpumalanga	Tree	Least concern	Fruit is eaten raw, used as a snack	Sugar, moisture, crude fat, protein, carbohydrate, ash, crude fiber, N, P, K, S, Mg, Ca, Zn, Fe, Mn, Cu	[14,15,35,106,129]
*Bridelia mollis* Hutch. **Phyllanthaceae**	Mokokwele (TW), Velvet sweet berry (E), Mokamanawa (TW), Fluweel-soetbessie (A)	Gauteng, Limpopo, and Mpumalanga	Shrub	Least concern	Sweet preserve, consumed as a snack	Not specified	[6,30,53,65]
*Flueggea virosa* (Roxb. ex Willd.) Voigt subsp. Virosa **Phyllanthaceae**	Umyaweyawe (Z), White-berry bush (E), Motlatlane (TW), Mutangauma (V)	Gauteng, KwaZulu-Natal, Limpopo, Mpumalanga, and North West	Tree	Least concern	Fruit is edible	Not specified	[14,30,37,40]
*Bechemia discolor* (Klotzsch) Hemsl. **Rhamnaceae**	Brown ivory (E), mewee (A), mogokgomo (Ns), Umzinzila (Nd), Nmumu (Z)	Limpopo and Mpumalanga	Tree	Least concern	Fruit is eaten raw, used to produce alcohol, used to make porridge	Ca, Fe, K, P, Mg, vitamin A, vitamin C	[41,130,131,132,133,134]
*Ziziphus mucronata* Willd. **Rhamnaceae**	Umlahlankosi (Z), Umphafa (ND), Wait-a-bit (E), Umphafa (X), Wag-’n-bietjie (A)	Eastern Cape, Free State, Gauteng, KwaZulu-Natal, Limpopo, Mpumalanga, Northern Cape, and North West	Tree	Least concern	Consumed raw	Vitamin C, beta carotene, ash, moisture content, fiber, fat, phytate, Ca, Fe, K, P, Mg	[41,131,132,135]
*Canthium inerme* (L.F.) Kuntze **Rubiaceae**	Turkey berry (E), umvuthwemini (Z), wolwedoring (A), muvhibvela-shadani (V)	Eastern Cape, Gauteng, KwaZulu-Natal, Limpopo, Mpumalanga, North West, and Western Cape	Tree	Least concern	Fruit is edible, snack	Moisture, fat, sugar, protein	[14,31,35,37,102]
*Vangueria infausta* Burch. **Rubiaceae**	Wildemispel (A), Muzwilu (V), Ntswila (T), Velvet Wild-medlar (E)	Eastern Cape, Free State, Gauteng, KwaZulu-Natal, Limpopo, Mpumalanga, Northern Cape, and North West	Tree	Least concern	Fruit is edible raw (dried), used to produce juice, apple sauce, alcoholic beverages, vinegar, jams, and pudding	Dry matter, protein, fat, carbohydrates, fiber, ash, Ca, Fe, K, P, Mg	[14,15,46,49,50,77,130,136,137,138,139]
*Dovyalis caffra* (Hook.f. & Harv.) Warb. **Salicaceae**	Kei-appel (A), Wild apricot (E), Umqokolo (ND), Muvhamba-nguvho (V)	Eastern Cape, KwaZulu-Natal, Limpopo, Mpumalanga and Western Cape	Tree	Least concern	Eaten raw, used to produce jam, jellies, juices, sweet and savory preserves, and wines	Water, energy, carbohydrates, ash, fiber, fat, protein, moisture, Fe, Cu, Zn, K, N, vitamin C	[30,33,61,140,141,142]
*Pappea capensis* Eckl. & Zeyh. **Sapindaceae**	Jacket-plum (E), doppruim (A); indaba (Z); ilitye (X); mongatane (S); liletsa (W), gulaswimbi (XT), umqhoqho	Eastern Cape, Free State, Gauteng, KwaZulu-Natal, Limpopo, Mpumalanga, Northern Cape, North West, and Western Cape	Tree	Least concern	Consumed as a snack, used to make tea, sweet and savory preserves, and produce alcoholic beverages	Carbohydrates, fiber, ash, fat, Fe, moisture, vitamin C	[30,32,33,37,59,65,106,143,144]
*Inhambanella henriquesii* (Engl. & Warb.) Dubard **Sapotaceae**	Milk-pear (E)	KwaZulu-Natal	Tree	Least concern	Consumed as a snack	Not specified	[30,33,40]
*Manilkara discolor* (Sond.) J.H.Hemsl. **Sapotaceae**	Forest milkberry (E)	KwaZulu-Natal	Tree	Least concern	Consumed as a snack	Not specified	[30,33,38,53]
*Mimusops zeyheri* Sond. **Sapotaceae**	Transvaal red milkwood (E), Umbumbulu (ND), Mububulu (V), Moepel (A)	KwaZulu-Natal, Limpopo, and Mpumalanga	Tree	Least concern	Consumed as a snack, used to produce alcoholic and non-alcoholic beverages	Organic matter, dry matter, carbohydrates, ash content, protein, Ca, P, Mg, vitamin E	[14,30,61,106,145,146,147,148]
*Vitellariopsis dispar* (N.E.Br.) Aubrév. **Sapotaceae**	Tugela bush-milkwood (E), Tugelabosmelkhout (A), Umphumbulu (Z)	KwaZulu-Natal	Tree	Rare	Consumed as a snack	Not specified	[149]
*Lantana rugosa* Thunb. **Verbenaceae**	Benyoni (Z), Bentaka (X), Molutoane (SS), Wildsalie (a), Bird’s beer (E)	Eastern Cape, Free State, Gauteng, KwaZulu-Natal, Limpopo, Mpumalanga, Northern Cape, North West, and Western Cape	Shrub	Least concern	Fruit is edible	Not specified	[14,30,37,52]
*Rhoicissus tridentata* (L.F) Wild & R.B. Drumm. **Vitaceae**	Bushman’s grape (E), Wild grape (E), Ulatile (X), Lumbu (XT), Wildedruif (A)	Eastern Cape, Free State, Gauteng, KwaZulu-Natal, Limpopo, Mpumalanga, North West, and Northern Cape	Climber	Least concern	Consumed as a snack, used to make jams, jellies, and wine	Not specified	[30,37,52,66,150,151]

Common names: English (E), Afrikaans (A), Venda (V), Xhosa (X), Ndebele (ND), Sesotho (S), Siswati (SW), Xitsonga (XT), and Sepulana (PL). Minerals: calcium (Ca), magnesium (Mg), phosphorus (P), manganese (Mn), nickel (Ni), iron (Fe), zinc (Zn), nitrogen (N), chromium (Cr), copper (Cu), aluminum (Al), chlorine (Cl), zirconium (Zr), molybdenum (Mo), mercury (Hg), cobalt (Co), boron (B), potassium (K), titanium (Ti), zirconium (Zr), strontium (Sr), and vitamins (V).

### 3.3. The Growth Habits of Wild Fruits

**Figure 3:**
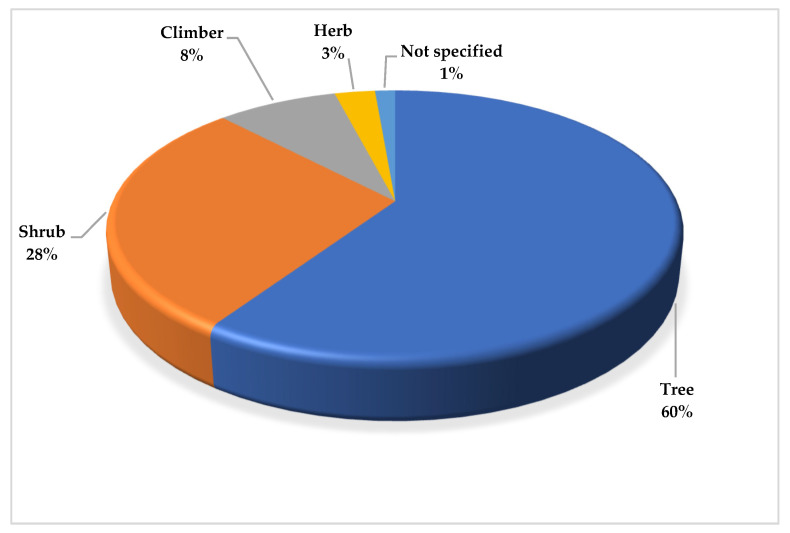
The growth habits of wild fruits.

Regarding the growth habits of the wild fruits in this current study, the documented wild plants had four life forms, i.e., trees, shrubs, climbers, and herbs. The trees were the most dominant, which comprised 44 species, accounting for 60% of the documented species, as stipulated in Figure 3 below, followed by the shrubs (n = 21) (28%), climbers (n = 6) (8%), and the least, the herbs (n = 2) (3%). Consequently, one out of the 74 documented species does not have a specified growth form i.e., *Hydrona africana* Thumb. These results aligns with those that were found in the study by Ramachandran [152], whereby trees were the most dominant growth form of wild fruits. In contrast, Asfaw, Lulekal [153], in their study, found that shrubs were the most dominant, followed by trees.

### 3.4. The Nutritional Composition and Uses of Some of the Key Wild Fruits in South Africa

Wild fruits are known to have nutritional and medicinal value; however, this review was only limited to their uses as a food source. Globally, wild fruit trees are gaining increasing recognition and importance due to their nutritional components [154]. Wild fruits serve as a source of essential nutrients, including both macro- and micronutrients, that are crucial in our diets [155]. The nutritional contents of wild fruits are very significant as they provide supplemental sustenance and vital nutrients, especially for local people who are mainly dependent on wild foods as they widely assist in food insecurity and malnutrition [156]. Incorporating wild fruits into our diets will exhibit higher diversity and greater nutrient quality as compared to cultivated fruits [157], as wild fruits have been endorsed as abundant sources of antioxidants, minerals, and vitamins [158,159]. Consequently, there has been an increasing interest to assess the nutritional characteristics of numerous wild fruits [160,161,162,163]. Scientific research has confirmed that certain fruits are advantageous suppliers of nutrients [77,164,165]. As noted by Kucich and Wicht [166], wild fruits in different parts of South Africa serve as an affordable and viable option for fulfilling our daily nutritional needs. Only 41 of the fruits that are tabulated have nutritional content information.

**Figure 4:**
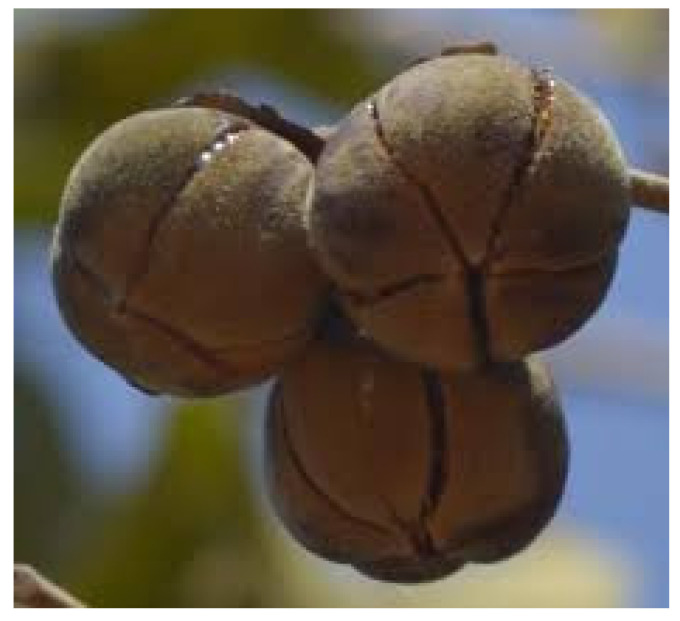
The *Azanza garckeana* (F.Hoffm.) Exell & Hillc. fruit. https://encrypted-tbn0.gstatic.com/images?q=tbn:ANd9GcRwA_ZruD7C9fOk78mDjvB39WsoG2-BdyHt4g&s (accessed on 18 April 2025).

*Azanza garckeana* (F.Hoffm.) Exell & Hillc. is a wild edible fruit species that falls under the Malvaceae family. It is commonly known as snot apple, muthowa, or jakjak and it is widely distributed in the Limpopo province. It can be consumed raw as a snack, or it can be used to make porridge. Figure 4 below shows the *Azanza garckeana* (F.Hoffm.) Exell & Hillc. fruits. They consists of the following mineral composition and proximate composition: Ca (9.5 mg/100 g), Fe (8.4 mg/100 g), K (2619 mg/100 g), P (147.6 mg/100 g), Mg (145.6 mg/100 g), Na (20.02 mg/100 g), fiber (45.3%), carbohydrates (35.2%), ash, moisture, proteins (12%), and fat (1%) [77].

*Annona senegalensis* Pers. is widely known as the “wild custard apple” and it belongs to the Annonaceae family. It is widely distributed in KwaZulu-Natal, Limpopo, and Mpumalanga. It contains 25.3% carbohydrates, 12.20% moisture, 24.0% fat, 12.10% ash, and 8.80% protein [56]. Ca (1.35 mg/g), K (0.47 mg/ g), Zn (0.48 mg/ g), Fe (1.80 mg/g), and Mn (0.13 mg/ g) are some of the mineral components that are found in the fruits of *A. senegalensis* [56]. It forms an important food source due to its nutritional composition.

**Figure 5:**
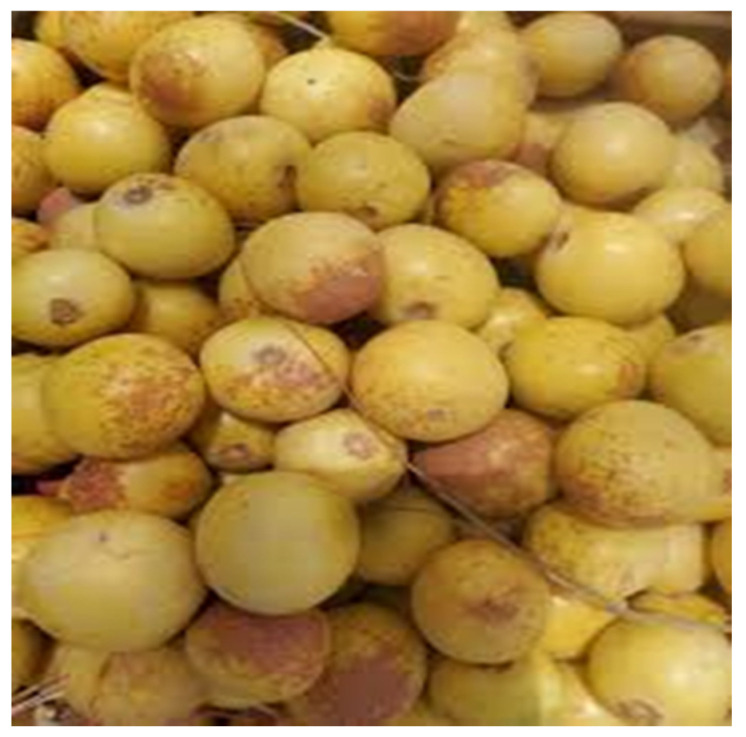
The marula fruit. https://encrypted-tbn0.gstatic.com/images?q=tbn:ANd9GcRT809YeV3HSUwZAvebgy2CunwB1y4_3xYQlQ&s (accessed on 18 April 2025).

*Sclerocarya birrea* Hochst. (marula fruit—as shown in Figure 5) is one of the most important wild fruits found in South Africa as declared by the Department of Agriculture, Forestry, and Fisheries. Consequently, it was selected for domestication and commercialization to enhance the nutritional status and welfare of people living in rural communities, especially during dry seasons [167]. It is a fruit tree that belongs to Anacardiaceae and is widely distributed in Gauteng, KwaZulu-Natal, Limpopo, Mpumalanga, and North West. The marula fruit is a rich source of vitamin C, which ranges from 62 mg/100 g to about 2100 mg/100 g [43,168,169,170]. It is distinguished as a commercially viable wild fruit, and this is supported by Moyo, Kulkarni [171], as they stipulated that the marula fruit is a food crop in several African countries and it is in huge demand for industrial uses. Vitamin C, which is also known as ascorbic acid, plays a crucial role in in enhancing food and nutritional security due to its numerous health advantages and contributions to general well-being, since humans cannot synthesize vitamin C, making its dietary intake from wild fruits necessary [172]. The marula fruit, as shown in Table 1, also consists of proteins, carbohydrates, dietary fiber, etc. According to Pfukwa, Chikwanha [25], the marula fruit significantly enhances nutrient consumption and serves a crucial function in our diets.

The marula fruit can be consumed raw or it can be used to produce products such as jelly, jam, juice, desserts, beer, chutneys, pie fillings, and sauces [173,174,175]; these products are sold in national and international markets, especially the Amarula liquor. According to Ndabikunze, Masambu [176], the fruits of *Sclerocarya birrea* can substantially enhance food and nutrition security at a household level. However, these fruits are underutilized even though they have the potential for reducing food and nutritional insecurity [177,178,179,180].

**Figure 6:**
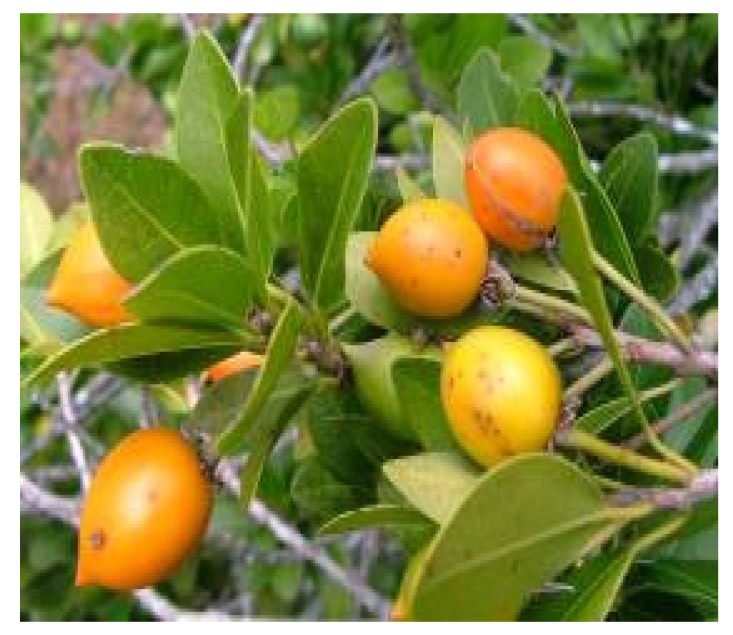
*Mimusops zeyheri*. https://pza.sanbi.org/sites/default/files/images/plants/10599/mimusopzeyfruit.jpg (accessed on 17 April 2025).

*Mimusops zeyheri* Sond., which has common names such as Transvaal red milkwood, (English), umpushane (Zulu), and mgamba kapu (Swati), is a perennial fruit tree belonging to the Sapotaceae family, its fruits are yellow orange, and it is also one of the most important wild fruits found in South Africa (Figure 6). Transvaal red milkwood fruits are a rich source of vitamins, proteins, and fatty acids as compared to commercial fruits such as guava, apples, and oranges, making them valuable sources to be added to our diets. According to Wilson and Downs [35], the fruits of *Mimusops zeyheri* contain higher levels of carbohydrates. Consequently, they demonstrate high amounts of ash content, proteins, starch, organic matter, dry matter, and carbohydrates, accounting for 2.80–4.1%, 9.30%, 83.30%, 91.10%, and 2.0%, respectively [147,148,181,182]. The vitamin C found in these fruits is higher than that typically found in cultivated fruits, accounting for 90 mg/100 g [183]. According to Mashela and Mollel [184], *Mimusops zeyheri* is one of the most fresh edible fruits. Moreover, Akinola, Pereira [8] highlighted that *Mimusops* zeyheri have the potential to reduce food and nutritional insecurity. Currently, they are used to produce beverages (alcoholic and non-alcoholic) and jellies, which are found in rural and urban open markets [146,185]. An example of this fruit is shown below.

*Strychnos spinosa* Lam., which is commonly known as the spiny monkey orange (E), belongs to the Loganiaceae family. In South Africa, it is widely distributed in the Eastern Cape, KwaZulu-Natal, Limpopo, and Mpumalanga Provinces. As noted in Table 1, the fruits of *Strychnos spinosa* consist of carbohydrates (42–60%), dry matter (19.7%), proteins (3.3%), moisture (74.6%), fat (2–31%), fiber (2.1%), ash (4.6%), Cu (0.04 mg/100 g–0.24 mg/100g), Mn (2.74 mg/100 g), K (1370 mg/100 g), Zn (0.22 mg/100 g), Ca (56 mg/100 g), Mg (49 mg/100 g), Na (21.7 mg/100 g), Fe (0.11 mg/100 g), P (66 mg/100 g), vitamin C (20 mg/100 g–88 mg/100 g), and energy (1923 KJ/100 g), making it have a significant nutritional value in rural populations [77,108,112,186]. The fruits of this plant are highly nutritious, and they contribute significantly to the diets of local people in South Africa. Among all other functions, *Strychnos spinosa* fruits serve as a source of income since some people are selling them [14,187,188]. However, the fruits of these species are limited to local consumption and have not entered commercial markets. The fruits of *Strychnos spinosa* are edible and are used to produce alcoholic and non-alcoholic beverages, i.e., they are recognized for their nutritional value. In contrast, these fruits are considered underutilized and they have a huge potential for contributing to sustainable food and nutrition [189].

**Figure 7:**
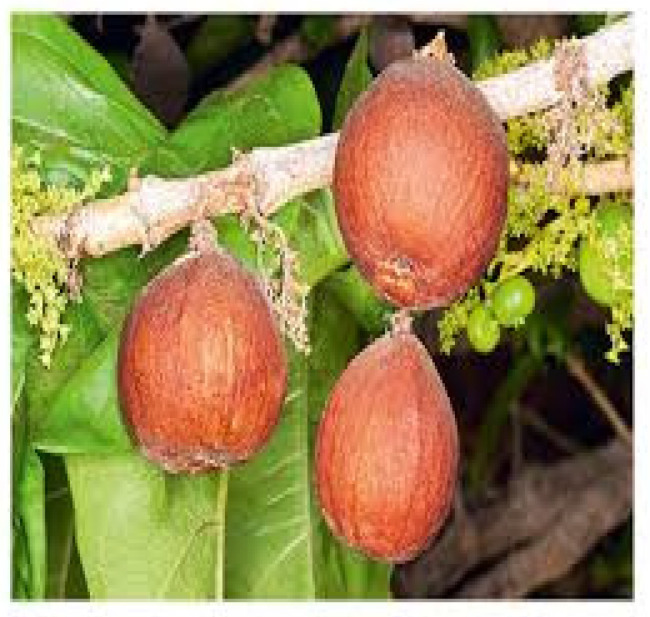
Monkey orange. https://encrypted-tbn0.gstatic.com/images?q=tbn:ANd9GcSh4YS5OS3P6XM9R86rFDtOazVSN91gajfxSQ&s (accessed on 17 April 2025).

*Vangueria infausta* Burch, which is commonly known as the African medlar, belongs to the Rubiaceae family. The Rubiaceae family fruit species, including *Vangueria infausta*, are rich in essential minerals such as Fe (0.09 mg/100 g–21.60 mg/100 g), Mg (0.06 mg/100 g–99.00 mg/100 g), and K (1.80 mg/100 g–1683.00 mg/100 g) [112,136,190]. According to Sibiya, Kayitesi [59], the essential minerals that are required by humans on a daily basis are prominently found in wild fruits. Hence, they are considered to be a very important food in the field of food science and technology [191]. Seemingly, Shai, Ncama [14] stated that wild fruit species have the capacity to enhance the food production sector, and they may contribute to the achievement of some of the sustainable development goals. However, there is limited research on the development and enhancement of wild fruit species [155]. *Vangueria infausta* is a widespread fruit plant that is native to South Africa. It is a widely consumed indigenous fruit among rural communities in South Africa. The fruits are rich in nutrients (macro and micro), as stipulated in Table 1. The African medlar fruit is mainly eaten raw, however, it can be used to produce products such as jam, juice, pudding, vinegar, and apple sauce, and it can be fermented to make alcoholic beverages such as beer and brandy [50,138]. According to Maroyi [192], the fruits of *Vangueria infausta* Burch have a huge potential for being commercialized as novel foods. Research by Steel and Behr [193] reported that some households use the juice of the fruit to enhance the flavor of porridge. As noted by Ráice [194], some of the fruits are dried for future uses, especially when there is food scarcity. Amarteifio and Mosase [112] highlighted that the fruits of *Vangueria infausta* Burch are a very good source of potassium, calcium, phosphorus, and magnesium as compared to some cultivated fruits (Figure 7).

**Figure 8:**
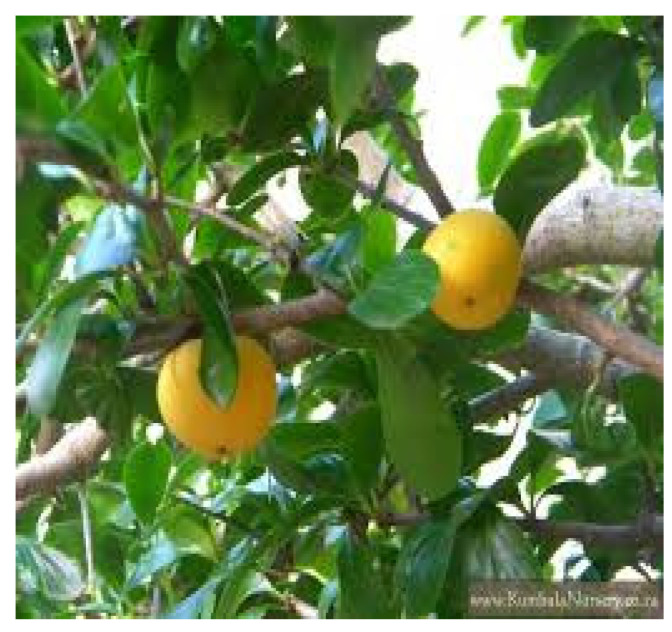
Kei apple. https://encrypted-tbn0.gstatic.com/images?q=tbn:ANd9GcQG-uFh37HuCPMq0-oMy4UeVl8D7_Zx3_LH4g&s (accessed on 17 April 2025).

*Dovyalis caffra* (Hook.f. & Harv.) Warb. is known as the “Kei apple” wild fruit species that belongs to the genus Dovyalis under the family Salicaceae. In South Africa, it is commonly found in Eastern Cape, KwaZulu-Natal, Limpopo, Mpumalanga, and Western Cape. Figure 8 depicts this fruit.

It consists of the following proximate composition, i.e., carbohydrates, moisture, protein, fiber, and ash which comprises 54.05%, 15.50%, 4.0%, 16.03%, and 7.45%, respectively [142]. Moreover, the fruits also contain Cu (0.06 mg/100 g), Mg (0.4 mg/100 g), K (606 mg/100 g), P (10.5 mg/100 g), Ca (4.8 mg/100 g), and Na (9.5 g/100 g) [126]. These fruits can be consumed fresh or they can be processed into products such as jams, jellies, juices, sweets, savory foods, preserves, and wines [195].

**Figure 9:**
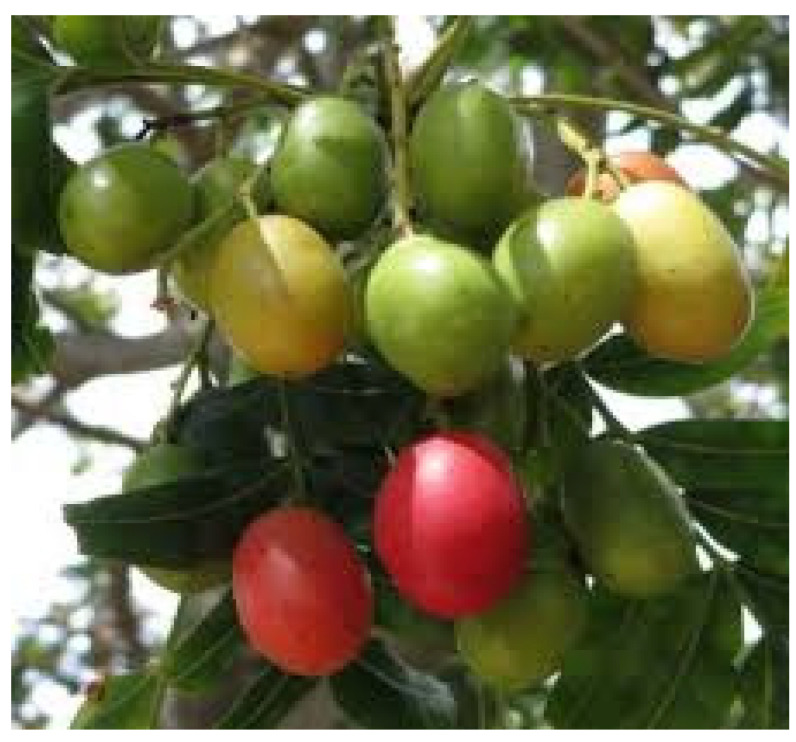
*Harpephyllum caffrum* Bernh. https://encrypted-tbn0.gstatic.com/images?q=tbn:ANd9GcS4hLzY2zyYyhLK4naQYKyIJBSqsE2_bcYH4Q&s (accessed on 17 April 2025).

*Harpephyllum caffrum* Bernh., commonly known as wild plum, belongs to one of the largest families, i.e., Anacardiaceae, and it is the only species that is recognized under this family. It spreads through Eastern Cape, KwaZulu-Natal, Limpopo, and Mpumalanga. It can be consumed raw as a snack, and used to produce jams, jellies, alcoholic and non-alcoholic beverages, and rose wines. Protein (0.7 g/100 g), fat (0.2 g/100 g), carbohydrates (9.1 g/100 g), ash (0.8 g/100 g), Ca (47.0 mg/100 g–115.8 mg/100 g), Cu (0.1 mg/100 g–0.4 mg/100 g), Fe (0.6 mg/100 g–2.9 mg/100 g), Mg (23.7 mg/100 g–26.4 mg/100 g), and vitamin C (70.7 mg/100 g) are some of the nutritional components of this wild fruit [196]. Figure 9 below depicts this fruit.

**Figure 10:**
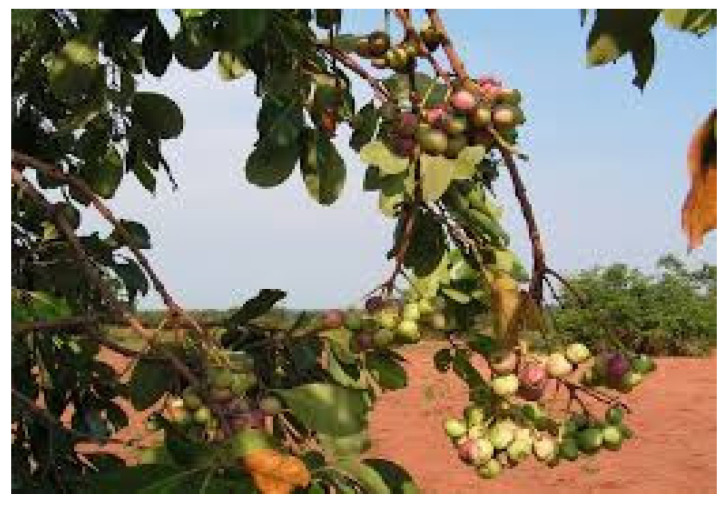
*Syzygium guineense* (Willd) DC. fruit. https://encrypted-tbn0.gstatic.com/images?q=tbn:ANd9GcQ1nFFG5372tcFCRkN9Zl7VHST3S5iXzlFQAg&s (accessed on 17 April 2025).

*Syzygium guineense* (Willd) DC. is an edible wild fruit species that is found across KwaZulu-Natal, Limpopo, and Mpumalanga in South Africa. It belongs to the Myrtaceae family, and it is commonly addressed as bushveld water berry and water pear. Figure 10 below represents this fruit. As stipulated by Maregesi, Kagashe [127], the fruit contains of the following mineral contents: Mn (8.5 mg/100 g), Ca (20.477 mg/100 g), Fe (268.3 mg/100 g), K (443 mg/100 g), P (8392 mg/100 g), and vitamin A (1.7 mg/100 g). It also consists of ash (3.34 g/100 g) moisture (82.4 g/100 g), proteins (1.66 g/100 g), carbohydrates (1.01 g/100 g), fat (7.74 g/100 g), and energy (80.34 g/100 g) [126]. According to Low, Rajaraman [197], the fruits of this species have great nutritional components and they can be used widely for making jams, drinks, and jellies.

### 3.5. Conservation Status

**Figure 11:**
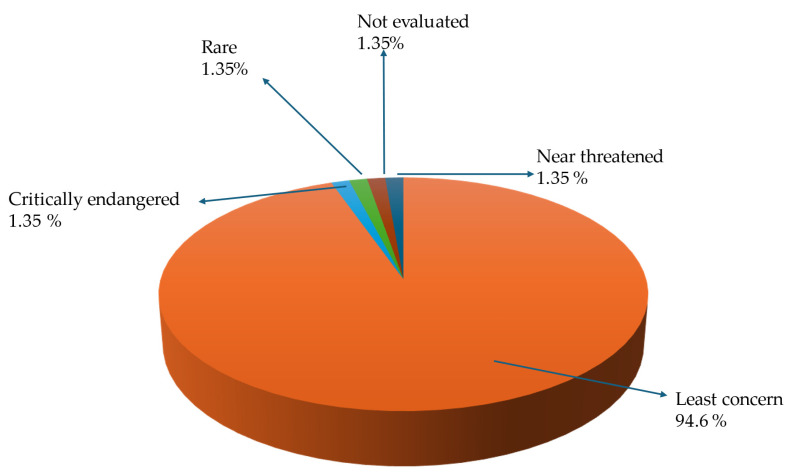
Conservation status of wild edible fruit species found in South Africa.

South Africa is a country that is rich in wild foods such as wild fruits. As in the study by Semenya and Mokgoebo [198], all the plant species that are documented in this study are listed in the South African National Red Data List of plants. In this current study, 94.56% of the documented wild fruits are classified as “least concern”, meaning that they are not threatened, whilst “critically endangered”, “rare”, “not evaluated”, and “near threatened” are each represented by one fruit species, which make up 1.35% of the total documented wild fruit species. These statuses of the documented wild fruit species were taken from the Red List of South African Plants. According to [199], species that are considered as rare are those species that naturally occur in a limited geographical region, inhabit one or a few specialized environments, and constitute only a small population within their distribution; in this case, the *Vitellariopsis dispar* (N.E.Br.) Aubrév. is only found in KwaZulu-Natal, and it is endemic to South Africa. Additionally, *Opuntia ficus-indica* (L.) Mill. (Figure 2) has not yet been evaluated for its conservation status, meaning that it has not yet been assessed against the criteria established by the International Union for Conservation of Nature (IUCN). As stated by Moraswi, Bamigboye [200], the evaluation of the conservation status of native plants is crucial, as threats to these species may adversely affect the natural ecosystem. For this present study, *Acanthosicyos horridus* Welw. ex Hook.f. is the only plant species that falls under the critically endangered species list, and *Cryptocarya wyliei* Stapf is the only nearly threatened plant species, as indicated in Figure 11.

## 4. Conclusions

This review compiled some of the nutritious edible wild fruits in South Africa. It further highlights the contribution of wild edible fruits to enhancing food and nutrition security countrywide. Studies of this stature are crucial for encouraging the development of grassroots food products while strengthening the fight against starvation, malnutrition, and food insecurity. Therefore, it is arguable that this study aligns with the sustainable development goal of zero hunger and food security. A total of 74 wild edible fruit species belonging to 29 families were inventoried with their dietary contents. However, this review has identified a dearth of information regarding the complete nutrition contents of some wild edible fruit species, including *Grewia flavor*, *Ficus burkei*, *Ficus petersii*, *Ficus sansibarica*, *Syzygium intermedium*, *Olea capensis*, *Bridelia mollis*, *Flueggea virosa*, *Manilkara discolor*, *Inhambanella henriquesii*, *Vitellariopsis dispan*, *Lantana rugosa*, *Rhoicissus tridentata*, *Lannea schweinfurthii*, *Ozoroa dispar*, *Searsia dentata*, *Searsia discolor*, *Searsia leptodictya*, *Searsia pendulina*, *Searsia undulata*, *Searsia pentheri*, *Hexalobul monopetalus*, *Ancylobotrys capensis*, *Pollichia campestris*, *Mystroxylon aethiopicum*, *Euclea crispa*, *Euclea divinorum*, *Euclea natalensis*, *Dialium schlechteri*, *Macrotyloma maranguense*, *Romulea rosea*, and *Cryptocarya wyliei*. In this regard, the current study recommends that future studies on wild edible fruits focus on nutrition content and new food development. Wild edible fruit species, such as *Sclerocarya birrea* (marula), *Mimusops zeyheri* (Transvaal red milkwood), and *Strychnos spinosa* (spiny monkey orange), were noted for their substantial contributions to dietary diversity, providing vitamins, minerals, proteins, etc. In addition, wild edible fruits could potentially enhance food and nutritional security in rural and marginalized South African communities. Furthermore, socio-economic studies, including the prospects of wild edible fruits’ potential to enhance new rural income generation streams, could give insights required to evaluate their economic contribution in rural areas.

## Data Availability

The original contributions presented in the study are included in the article; further inquiries can be directed to the corresponding authors.

## References

[1] Rowe A.K., Hirnschall G., Lambrechts T., Bryce J. (1999). Linking the integrated management of childhood illness (IMCI) and health information system (HIS) classifications: Issues and options. Bull. World Health Organ..

[2] Berry E.M., Dernini S., Burlingame B., Meybeck A., Conforti P. (2015). Food Security and Sustainability: Can One Exist Without the Other?. Public Health Nutr..

[3] Food and Agriculture Organization (FAO) (2017). The State of Food Security and Nutrition in the World 2017: Building Resilience for Peace and Food Security.

[4] Grant M. (2015). A Food Systems Approach for Food and Nutrition Security. Sight Life.

[5] El Bilali H., Callenius C., Strassner C., Probst L. (2019). Food and Nutrition Security and Sustainability Transitions in Food Systems. Food Energy Secur..

[6] Van Wyk B.-E. (2011). The Potential of South African Plants in the Development of New Food and Beverage Products. S. Afr. J. Bot..

[7] Welcome A., Van Wyk B.-E. (2019). An Inventory and Analysis of the Food Plants of Southern Africa. S. Afr. J. Bot..

[8] Akinola R., Pereira L.M., Mabhaudhi T., De Bruin F.M., Rusch L. (2020). A Review of Indigenous Food Crops in Africa and the Implications for More Sustainable and Healthy Food Systems. Sustainability.

[9] Borelli T., Hunter D., Powell B., Ulian T., Mattana E., Termote C., Pawera L., Beltrame D., Penafiel D., Tan A. (2020). Born to eat wild: An integrated conservation approach to secure wild food plants for food security and nutrition. Plants.

[10] Shackleton C., Shackleton S. (2004). The Importance of Non-Timber Forest Products in Rural Livelihood Security and as Safety Nets: A Review of Evidence from South Africa. S. Afr. J. Sci..

[11] Tebkew M., Gebremariam Y., Mucheye T., Alemu A., Abich A., Fikir D. (2018). Uses of Wild edible Plants in Quara District, Northwest Ethiopia: Implication for Forest Management. Agric. Food Secur..

[12] Hazarika T.K., Varte L., Mathipi V., Khawlhring L., Lalruatsangi E., Debbarma P., Senthil Kumar N. (2023). Phytochemicals Constituents, Antioxidant Activities and Cytotoxicity Assays of Few wild Edible Fruits of North-East India. Int. J. Food Prop..

[13] Shamseer L. (2016). Preferred reporting items for systematic review and meta-analysis protocols (PRISMA-P) 2015: Elaboration and explanation. BMJ.

[14] Shai K.N., Ncama K., Ndhlovu P.T., Struwig M., Aremu A.O. (2020). An Exploratory Study on the Diverse Uses and Benefits of Locally-Sourced Fruit Species in Three Villages of Mpumalanga Province, South Africa. Foods.

[15] Mashile S., Tshisikhawe M., Masevhe N. (2019). Indigenous Fruit Plants Species of the Mapulana of Ehlanzeni District in Mpumalanga province, South Africa. S. Afr. J. Bot..

[16] Maroyi A. (2011). The gathering and consumption of wild edible plants in Nhema communal area, Midlands province, Zimbabwe. Ecol. Food Nutr..

[17] Mitchell J.D., Pell S.K., Bachelier J.B., Warschefsky E.J., Joyce E.M., Canadell L.C., da Silva-Luz C.L., Coiffard C. (2022). Neotropical Anacardiaceae (cashew family). Braz. J. Bot..

[18] Cunha A., David J. (2024). Chemical Composition, Biological Activities and Uses of Anacardiaceae Species: An Updated Review. Química Nova.

[19] Clement W.L., Weiblen G.D. (2009). Morphological Evolution in the Mulberry Family (Moraceae). Syst. Bot..

[20] Gardner E.M., Sarraf P., Williams E.W., Zerega N.J. (2017). Phylogeny and Biogeography of Maclura (Moraceae) and the Origin of an Anachronistic Fruit. Mol. Phylogenetics Evol..

[21] Rohwer J.G., Berg C.C. (1993). Moraceae. Flowering Plants· Dicotyledons: Magnoliid, Hamamelid and Caryophyllid Families.

[22] Berg C. (1980). Moraceae. Flora Neth. Antill..

[23] Dejene T., Agamy M.S., Agúndez D., Martin-Pinto P. (2020). Ethnobotanical survey of wild edible fruit tree species in lowland areas of Ethiopia. Forests.

[24] Akinyede K.A., Ekpo O.E., Oguntibeju O.O. (2020). Ethnopharmacology, Therapeutic Properties and Nutritional Potentials of Carpobrotus edulis: A Comprehensive Review. Sci. Pharm..

[25] Pfukwa T.M., Chikwanha O.C., Katiyatiya C.L., Fawole O.A., Manley M., Mapiye C. (2020). Southern African Indigenous Fruits and Their Byproducts: Prospects as Food Antioxidants. J. Funct. Foods.

[26] De Vynck J., Van Wyk B.-E., Cowling R. (2016). Indigenous Edible Plant use by Contemporary Khoe-San Descendants of South Africa’s Cape South Coast. S. Afr. J. Bot..

[27] Broomhead N.K., Moodley R., Jonnalagadda S.B. (2020). Chemical and Elemental Analysis of the Edible Fruit of Five Carpobrotus species from South Africa: Assessment of Nutritional Value and Potential Metal Toxicity. Int. J. Environ. Health Res..

[28] Vila M., D’Antonio C.M. (1998). Fruit Choice and Seed Dispersal of Invasive vs. Noninvasive Carpobrotus (Aizoaceae) in Coastal California. Ecology.

[29] Yahia E.M. (2011). Postharvest Biology and Technology of Tropical and Subtropical Fruits: Fundamental Issues.

[30] Ackhurst A. (1996). Interactive Data Base on All Edible Fruits in Southern Africa.

[31] Dlamini B. (1981). Swaziland Flora: Their Local Names and Uses.

[32] Dold T., Cocks M. (2000). Indigenous Plant Use of the AmaXhosa People on the Eastern Border of the Great Fish River Reserve, Eastern Cape. Ann. East. Cape Mus..

[33] Homewood K., Peters C.R., O’Brien E.M., Drummond R.B. (1993). Edible Wild Plants of Sub-Saharan Africa. Kew Bull..

[34] Moodley R., Koorbanally N., Jonnalagadda S.B. (2012). Elemental Composition and Fatty Acid Profile of the Edible Fruits of Amatungula (*Carissa macrocarpa*) and Impact of Soil Quality on Chemical Characteristics. Anal. Chim. Acta.

[35] Wilson A.-L., Downs C. (2012). Fruit Nutritional Composition and Non-Nutritive Traits of Indigenous South African Tree Species. S. Afr. J. Bot..

[36] Maroyi A. (2019). Medicinal Uses, Biological and Chemical Properties of Wild Plum (*Harpephyllum caffrum*): An Indigenous Fruit Plant of Southern Africa. J. Pharm. Nutr. Sci..

[37] Magwede K., Van Wyk B.E., Van Wyk A.E. (2019). An inventory of Vhavenḓa useful plants. S. Afr. J. Bot..

[38] Fox F.W., Norwood Young M.E. (1988). Food From the Veld: Edible Wild Plants of Southern Africa Botanically Identified and Described.

[39] Archer F.M. (1994). Ethnobotany of Namaqualand: The Richtersveld.

[40] Wehmeyer A. (1986). Edible Wild Plants of Southern Africa: Data on the Nutrient Contents of Over 300 Species.

[41] Aganga A., Mosase K. (2001). Tannin content, nutritive value and dry matter digestibility of Lonchocarpus capassa, Zizyphus mucronata, Sclerocarya birrea, Kirkia acuminata and Rhus lancea seeds. Anim. Feed Sci. Technol..

[42] Mariod A.A., Abdelwahab S.I. (2012). *Sclerocarya birrea* (Marula), An African Tree of Nutritional and Medicinal Uses: A Review. Food Rev. Int..

[43] Mashau M.E., Kgatla T.E., Makhado M.V., Mikasi M.S., Ramashia S.E. (2022). Nutritional Composition, Polyphenolic Compounds and Biological Activities of Marula Fruit (*Sclerocarya birrea*) With its Potential Food Applications: A Review. Int. J. Food Prop..

[44] Kamanula M., Munthali C.Y., Kamanula J.F. (2022). Nutritional and Phytochemical Variation of Marula (*Sclerocarya birrea*) (subspecies caffra and birrea) Fruit Among Nine International Provenances Tested in Malawi. Int. J. Food Sci..

[45] Boon R. (2010). Pooley’s Trees of Eastern South Africa: A Dictionary of Plant Use and Application.

[46] Shackleton C.M., Dzerefos C.M., Shackleton S.E., Mathabela F.R. (2000). The Use of and Trade in Indigenous Edible Fruits in the Bushbuckridge Savanna Region, South Africa. Ecol. Food Nutr..

[47] Eromosele I.C., Eromosele C.O., Kuzhkuzha D.M. (1991). Evaluation of mineral elements and ascorbic acid contents in fruits of some wild plants. Plant Foods Hum. Nutr..

[48] Jaenicke H., Thiong’o M.K. Preliminary nutritional analysis of marula (*Sclerocarya birrea*) fruits from two Kenyan provenances. Proceedings of the II ISHS Conference on Fruit Production in the Tropics and Subtropics.

[49] Magaia T., Uamusse A., Sjöholm I., Skog K. (2013). Proximate Analysis of Five Wild Fruits of Mozambique. Sci. World J..

[50] Stadlmayr B., Charrondiere U.R., Eisenwagen S., Jamnadass R., Kehlenbeck K. (2013). Nutrient Composition of Selected Indigenous Fruits from Sub-Saharan Africa. J. Sci. Food Agric..

[51] Moffett R. (2010). Sesotho Plant and Animal Names and Plants Used by the Basotho.

[52] Moteetee A., Van Wyk B.-E. (2006). Sesotho Names for Exotic and Indigenous Edible Plants in Southern Africa. Bothalia.

[53] Wyk B.V., Gericke N. (2000). People’s Plants: A Guide to Useful Plants of Southern Africa.

[54] Koenen E.V. (2001). Medicinal Poisonous and Edible Plants in Namibia.

[55] Van Wyk B.-E., Gorelik B. (2017). The History and Ethnobotany of Cape Herbal Teas. S. Afr. J. Bot..

[56] Yisa J., Egila J.N., Darlinton A.O. (2010). Chemical Composition of Annona senegalensis from Nupe land, Nigeria. Afr. J. Biotechnol..

[57] Rabelo S.V., Quintans J.S.S., Costa E.V., Almeida J.R., Júnior L.J., Preedy V.R. (2016). Chapter-24: Annona species (Annonaceae) oils. Essential Oils in Food Preservation, Flavor and Safety.

[58] Kitadi J.M., Inkoto C.L., Lengbiye E.M., Tshibangu D.S.T., Tshilanda D.D., Ngbolua K.N., Taba K.M., Mbala B.M., Schmitz B., Mpiana P.T. (2020). Mineral Content and Antisickling Activity of *Annona senegalensis*, *Alchornea cordifolia* and *Vigna unguiculata* used in the Management of Sickle Cell Disease in the Kwilu Province (Congo, DR). Int. Blood Res. Rev..

[59] Sibiya N., Kayitesi E., Moteetee A. (2020). Mineral Composition of Selected Indigenous Wild Southern African fruits. S. Afr. J. Bot..

[60] Skead C.J. (2009). Historical Plant Incidence in Southern Africa: A Collection of Early Travel Records in Southern Africa.

[61] (2013). Department of Agriculture Forestry Fisheries Most Common Indigenous Food Crops of South Africa, S. Afr. J. Plant Soil.

[62] Magwede K. (2018). A Quantitative Survey of Traditional Plant Use of the Vhavenḓa, Limpopo Province, South Africa. Doctoral Dissertation.

[63] Siyum Z.H., Meresa T.A. (2021). Physicochemical Properties and Nutritional Values of *Carissa spinarum* L./“AGAM” Fruit. Int. J. Fruit Sci..

[64] Souilem F., Dias M.I., Barros L., Calhelha R.C., Alves M.J., Harzallah-Skhiri F., Ferreira I.C. (2019). Amantagula fruit (*Carissa macrocarpa* (Eckl.) A. DC.): Nutritional and Phytochemical Characterization. Plant Foods Hum. Nutr..

[65] Liengme C. (1981). Plants Used by the Tsonga People of Gazankulu. Bothalia.

[66] Mabogo D.E.N. (2012). The Ethnobotany of the Vhavenda. Masters Dissertation.

[67] Ramadan M.F., Mörsel J.T. (2003). Lipid Profile of Prickly Pear Pulp Fractions. J. Food Agric. Environ..

[68] Chiteva R., Wairagu N. (2013). Chemical and Nutritional Content of *Opuntia ficusindica* (L.). Afr. J. Biotechnol..

[69] Heath R., Heath A. (2009). Field Guide to the Plants of Northern Botswana Including the Okavango Delta.

[70] Homewood K., Peters C.R., O’Brien E.M., Drummond R.B. (1992). Edible Wild Plants of Subsaharan Africa.

[71] Ellery K., Ellery W. (1997). Plants of the Okavango Delta: A Field Guide. J. Food Nutr. Sci..

[72] Magaia T., Uamusse A., Sjöholm I., Skog K. (2013). Dietary Fiber, Organic Acids and Minerals in Selected Wild Edible Fruits of Mozambique. Springerplus.

[73] Magaia T. (2015). Chemical Analysis to Promote the Use of Wild Fruits from Mozambique. Doctoral Dissertation.

[74] Von Koenen E.M. (1996). Medicinal Poisonous and Edible Plants in Namibia.

[75] Baumgärtel C., Förster A., Frommherz L., Henle T., José Ramiro G., Afonso F., Lautenschläger T. (2022). Potential and Nutritional Properties of Local Food Plants from Angola to Combat Malnutrition—Suitable Alternatives to Frequently Cultivated Crops. J. Appl. Bot. Food Qual..

[76] Muchuweti M., Matongo N., Benhura M.A.N., Bhebhe M., Kasiyamhuru A., Chipurura B. Nutritional composition of *Parinari curatellifolia* fruit and a jam made from the pulp of the fruit: An untapped resource. Proceedings of the II International Symposium on Underutilized Plant Species: Crops for the Future-Beyond Food Security. Acta Hort.

[77] Saka J.K., Msonthi J.D. (1994). Nutritional Value of Edible Fruits of Indigenous Wild Trees in Malawi. For. Ecol. Manag..

[78] Benhura C., Benhura M.A.N., Muchuweti M., Nyagura S.F., Gombiro P.E. (2012). Proximate Analysis of Fruit Pulp of Fruit from Parts of Harare and a Rural Area in Zimbabwe. Pak. J. Nutr..

[79] Chatepa L.E.C., Masamba K., Jose M. (2018). Proximate Composition, Physical Characteristics and Mineral Content of Fruit, Pulp and Seeds of Parinari curatellifolia (Maula) from Central Malawi. Afr. J. Food Sci..

[80] Coates Palgrave K. (1977). Trees of Southern Africa. Veld Flora.

[81] Maundu M.P., Ngugi W.G., Kabuye H.S.C. (1999). Traditional Food Plants of Kenya.

[82] Kadanthottu S.J., Bolla S., Joshi K., Bhat M., Naik K., Patil S., Bendre S., Gangappa B., Haibatti V., Payamalle S. (2017). Determination of Chemical Composition and Nutritive Value with Fatty Acid Compositions of African Mangosteen (*Garcinia livingstonei*). Erwerbs-Obstbau.

[83] Van Damme P., Van Den Eynden V. (1992). Plant Uses by the Topnaar of the Kuiseb Valley Namib desert. Afr. Focus.

[84] Van Damme P., Van Den Eynden V. (2000). Succulent and Xerophytic Plants Used by the Topnaar of Namibia. Haseltonia.

[85] Wilkins-Ellert M. (2004). Acanthosicyos horridus Welw. ex Hook. f. Plant Resour. Trop. Afr..

[86] Silberbauer G., Tanaka J., Hughes D.W. (1980). The San Hunter-Gatherers of the Kalahari: A Study in Ecological Anthropology.

[87] Klopatek J.M., Stock W.D. (1994). Partitioning of Nutrients in Acanthosicyos horridus, A Keystone Endemic Species in the Namib Desert. J. Arid Environ..

[88] Velempini K., Perkins J.S. (2008). Integrating Indigenous Technical Knowledge and Modern Scientific Knowledge for Biodiversity Conservation and Human Livelihoods in the Southern Kalahari, Botswana. Botsw. Notes Rec..

[89] Anhwange B., Ikyenge B.A., Nyiatagher D.T., Ageh J.T. (2010). Chemical Analysis of *Citrullus lanatus* (Thunb.), *Cucumeropsis mannii* (Naud.) and *Telfairia occidentalis* (Hook F.) Seeds Oils. J. Appl. Sci. Res..

[90] Fila W.A., Itam E.H., Johnson J.T., Odey M.O., Effiong E.E., Dasofunjo K., Ambo E.E. (2013). Comparative Proximate Compositions of Watermelon Citrullus lanatus, squash Cucurbita pepo’l and Rambutan Nephelium lappaceum. Int. J. Sci. Technol..

[91] Manivannan A., Lee E.S., Han K., Lee H.E., Kim D.S. (2020). Versatile Nutraceutical Potentials of Watermelon—A Modest Fruit Loaded with Pharmaceutically Valuable Phytochemicals. Molecules.

[92] Wehmeyer A. (1966). The Nutrient Composition of Some Edible Wild Fruits Found in the Transvaal. S. Afr. Med. J..

[93] Roodt V. (1998). Trees and Shrubs of the Okavango Delta: Medicinal Uses and Nutritional Value.

[94] Odhav B., Beekrum S., Akula U.S., Baijnath H. (2007). Preliminary Assessment of Nutritional Value of Traditional Leafy Vegetables in KwaZulu-Natal, South Africa. J. Food Compos. Anal..

[95] Ferrara L. (2018). A Fruit to Discover: Cucumis metuliferus E. Mey Ex Naudin (Kiwano). Agric. Food Sci. J..

[96] Romero-Rodriguez M., Vazquez-Oderiz M.L., Lopez-Hernandez J., Simal-Lozano J. (1992). Physical and Analytical Characteristics of the Kiwano. J. Food Compos. Anal..

[97] Flyman M.V., Afolayan A.J. (2007). The Implication of the Mineral ratios of *Cucumis myriocarpus* Naud. and *Pergularia daemia* (Forsk.) Chiov. in Human Diets. J. Med. Food.

[98] Ebbo A.A., Sani D., Suleiman M.M., Ahmed A., Hassan A.Z. (2019). Phytochemical Composition, Proximate Analysis and Antimicrobial Screening of the Methanolic Extract of *Diospyros mespiliformis* Hochst Ex a. Dc (Ebenaceae). Pharmacogn. J..

[99] Ebbo A., Mammam M., Suleiman M.M., Ahmed A., Bello A. (2014). Preliminary Phytochemical Screening of *Diospyros mespiliformis*. Anat. Physiol.

[100] Nyambe M.M., Hakwenye H., Benyamen M.S. (2019). Nutritional and Anti-Nutritional Composition of *Diospyros mespiliformis* and *Hyphaene petersiana* fruits from Namibia. Int. Sci. Technol. J. Namib..

[101] Du Preez R. (2003). Fruits of Tropical Climates|Lesser-Known Fruits of Africa. J. S. Afr. J. Clin. Nutr..

[102] Corrigan B., Van Wyk B.E., Geldenhuys C.J., Jardine J.M. (2011). Ethnobotanical Plant Uses in the KwaNibela Peninsula, St lucia, South Africa. S. Afr. J. Bot..

[103] Bolin J.F., Tennakoon K.U., Maass E. (2010). Mineral Nutrition and Heterotrophy in the Water Conservative *Holoparasite hydnora* Thunb.(Hydnoraceae). Flora-Morphol. Distrib. Funct. Ecol. Plants.

[104] De Beer J.J., Van Wyk B.E. (2011). An Ethnobotanical Survey of the Agter–Hantam, Northern Cape Province, South Africa. S. Afr. J. Bot..

[105] Leffers A. (2003). Traditional Plant Use by Jul’hoansi in North-Eastern Namibia.

[106] Motlhanka D., Motlhanka P., Selebatso T. (2008). Edible Indigenous Wild Fruit Plants of Eastern Botswana. Int. J. Poult. Sci..

[107] Arnold T.H., Wells M.J., Wehmeyer A.S. Khoisan Food Plants: Taxa with Potential for Future Economic Exploitation. Proceedings of the Plants for Arid Lands: Proceedings of the Kew International Conference on Economic Plants for Arid Lands held in the Jodrell Laboratory, Royal Botanic Gardens.

[108] Omotayo A.O., Aremu A.O. (2021). Undervalued spiny Monkey Orange (*Strychnos spinosa* Lam.): An indigenous fruit for sustainable food-nutrition and economic prosperity. Plants.

[109] Oboh M., Zharare G., Osunsanmi F., Mosa R., Opoku A. (2023). Nutritional Composition and Cytotoxicity Studies of Black Monkey (Strychnos madagascariensis) Ripe Fruit. Afr. J. Food Agric. Nutr. Dev..

[110] Van Rayne K.K., Adebo O.A., Ngobese N.Z. (2020). Nutritional and Physicochemical Characterization of Strychnos madagascariensis Poir (Black Monkey Orange) Seeds as a Potential Food Source. Foods.

[111] Ngadze R.T., Linnemann A.R., Nyanga L.K., Fogliano V., Verkerk R. (2017). Local Processing and Nutritional Composition of Indigenous Fruits: The Case of Monkey Orange (Strychnos spp.) from Southern Africa. Food Rev. Int..

[112] Amarteifio J., Mosase M. (2006). The Chemical Composition of Selected Indigenous Fruits of Botswana. J. Appl. Sci. Environ. Manag..

[113] Sitrit Y., Loison S., Ninio R., Dishon E., Bar E., Lewinsohn E., Mizrahi Y. (2003). Characterization of Monkey Orange (*Strychnos spinosa* Lam.), A Potential New Crop for Arid Regions. J. Agric. Food Chem..

[114] Jacob C., Shehu Z.C., Danbature W.L., Karu E. (2016). Proximate Analysis of the Fruit *Azanza garckeana* (“Goron Tula”). Bayero J. Pure Appl. Sci..

[115] Nkafamiya I., Ardo B.P., Osemeahon S.A., Akinterinwa A. (2016). Evaluation of Nutritional, Non-nutritional, Eelemental Content and Amino Acid Profile of Azanza garckeana (Goron Tula). Br. J. Appl. Sci. Technol..

[116] Suliman A.M.E., Difa I.Y., Salih Z.A. (2012). The Nutritive Value of Jakjak (*Azanza garckeana* L.) Fruit and its Utilization in Juice Production. Asian J. Biol. Sci..

[117] Mojeremane W., Tshwenyane S. (2004). *Azanza garckeana*: A valuable Edible Indigenous Fruit Tree of Botswana. J. Biol. Agric. Healthc..

[118] Elhassan G.M., Yagi S.M. (2010). Nutritional Composition of Grewia species (*Grewia tenax* (Forsk.) Fiori, *G. flavescens* Juss and *G. villosa* Willd) fruits. J. Food Sci. Technol..

[119] Tsomele G.F., Venter E., Wokadala O.C., Jooste E., Dlamini B.C., Ngobese N.Z., Siwela M. (2021). Structural (Gross and Micro), Physical and Nutritional Properties of *Trichilia emetica* and *Trichilia dregeana* Seeds. CyTA-J. Food.

[120] Norwood Young M.E., Fox F.W. (1982). Food from the Veld–Edible Wild Plants Found in the Kalahari.

[121] Lewis W.H. (1986). The Ethnobotany of the Kwanyama Ovambos. Monographs in Systematic Botany.

[122] Acipa A., Kamatenesi-Mugisha M., Oryem-Origa H. (2013). Documentation and Nutritional Profile of Some Selected Food Plants of Otwal and Ngai Sun Counties Oyam District, Northern Uganda. Afr. J. Food Agric. Nutr. Dev..

[123] Saloufou K.I., Boyode P., Simalou O., Eloh K., Idoh K., Melila M., Toundou O., Kpegba K., Agbonon A. (2018). Chemical Composition and Antioxidant Activities of Different Parts of Ficusur. J. Herbmed Pharmacol..

[124] Roodt V. (1992). The Shell Field Guide to the Common Trees of the Okavango Delta and Moremi Game Reserve.

[125] Maliehe S.T. (2015). An Evaluation of Nutraceutical Components of Syzygium cordatum Fruits for the Treatment of Gastrointestinal Tract Infections.

[126] Sibiya N.P., Kayitesi E., Moteetee A.N. (2021). Proximate Analyses and Amino Acid Composition of Selected Wild Indigenous Fruits of Southern Africa. Plants.

[127] Maregesi S., Kagashe G., Messo C.W., Mugaya L. (2016). Determination of Mineral Content, Cytotoxicity and Anthelmintic Activity of *Syzygium guineense* Fruits. Saudi J. Med. Pharm. Sci..

[128] Fox F.W., Norwood Young M.E. (1988). Food from the Veld: Edible Wild Plants of Southern Africa Botanically Identified and Described.

[129] Murthy H.N., Dalawai D., Mamatha U., Angadi N.B., Dewir Y.H., Al-Suhaibani N.A., El-Hendawy S., Al-Ali A.M. (2021). Bioactive Constituents and Nutritional Composition of *Bridelia stipularis* L. Blume Fruits. Int. J. Food Prop..

[130] Ohiokpehai O. (2003). Promoting the Nutritional Goodness of Traditional Food Products. Pak. J. Nutr..

[131] Aganga A., Mesho E. (2008). Mineral Contents of Browse Plants in Kweneng District in Botswana.

[132] Ondiek J.O., Abdulrazak S.A., Njoka E.N. (2010). Chemical and Mineral Composition, In-Vitro Gas Production, In-Sacco Degradation of Selected Indigenous Kenyan Browses. Livest. Res. Rural Dev..

[133] Feyssa D.H., Njoka J.T., Asfaw Z., Nyangito M.M. (2012). Uses and Management of Ximenia americana, Olacaceae in Semi-Arid East Shewa, Ethiopia. Pak. J. Bot..

[134] Okoye J., Oni K. (2017). Promotion of Indigenous Food Preservation and Processing Knowledge and the Challenge of Food Security in Africa. J. Food Secur..

[135] Tsegaye M., Alemu T., Dilnessa A., Tolessa A., Tantu T., Bekalu Y., Haile F. (2023). Effect of Storage Condition on the Nutritional and Anti-Nutritional Composition of Kurkura (*Ziziphus mauritiana* Lam.) Fruit from North-Eastern Ethiopia. Heliyon.

[136] Mothapo M.J. (2014). Physico-Chemical Properties and Selected Nutritional Components of Wild Medlar (*Vangueria infausta*) Fruit Harvested at Two Harvesting Time.

[137] Legwaila G., Mojeremane W., Madisa M.E., Mmolotsi R.M., Rampart M. (2011). Potential of Traditional Food Plants in Rural Household Food Security in Botswana. J. Hortic. For..

[138] Sofowora A., Ogunbodede E., Onayade A. (2013). The Role and Place of Medicinal Plants in the Strategies for Disease Prevention. Afr. J. Tradit. Complement. Altern. Med..

[139] Norwood Young M., Fox F. (1982). Food From the Veld: Edible Wild Plants of Southern Africa Botanically Identified and Described.

[140] Aremu A.O., Ncama K., Omotayo A.O. (2019). Ethnobotanical Uses, Biological Activities and Chemical Properties of Kei-apple [Dovyalis caffra (Hook. f. & Harv.) Sim]: An Indigenous Fruit Tree of Southern Africa. J. Ethnopharmacol..

[141] Magwede K., Van Wyk B.-E. (2016). An Inventory of Vhavenda Useful Plants, Limpopo Province, South Africa. S. Afr. J. Bot..

[142] Taher M.A., Tadros L.K., Dawood D.H. (2018). Phytochemical Constituents, Antioxidant Activity and Safety Evaluation of Kei-apple fruit (*Dovyalis caffra*). Food Chem..

[143] Osuga I., Abdulrazak S.A., Nishino N., Ichinohe T., Fujihara T. (2006). Potential Nutritive Value of Selected Browse Species from Kenya Using in Vitro Gas Production Technique and Polyethylene Glycol. Livest. Res. Rural Dev..

[144] Karau G.M., Njagi E.N., Machocho A.K., Wangai L.N. (2012). Phytonutrient, Mineral Composition and In Vitro Antioxidant Activity of Leaf and Stem Bark Powders of *Pappea capensis* (L.). Pak. J. Nutr..

[145] Lubisi N.P., Ramarumo L.J., Manyaga M., Mbeng W.O., Mokgehle S. (2023). Perceptions on Utilization, Population, and Factors that are Affecting Local Distribution of Mimusops zeyheri in the Vhembe Biosphere Reserve, South Africa. Biodiversitas J. Biol. Divers..

[146] Chivandi E., Davidson B., Pretorius B., Erlwanger K. (2011). Proximate, Mineral, Amino Acid, Fatty Acid, Vitamin E, Phytate Phosphate and Fibre Composition of Mimusops zeyheri (Red Milkwood) Seed. Int. J. Food Sci. Technol..

[147] Mngadi S., Moodley R., Jonnalagadda S.B. (2017). Elemental composition and nutritional value of the edible fruits of coastal red-milkwood (*Mimusops caffra*) and impact of soil quality on their chemical characteristics. J. Environ. Sci. Health Part B.

[148] Chivandi E., Cave E., Davidson B.C., Erlwanger K.H., Moyo D., Madziva M.T. (2012). Suppression of Caco-2 and HEK-293 cell proliferation by *Kigelia africana*, *Mimusops zeyheri* and *Ximenia caffra* seed oils. Vivo.

[149] Smith C.A. (1966). Common Names of South African Plants. Bothalia J..

[150] Boon R. (2010). Pooley’s Trees of Eastern South Africa: Flora and Fauna Publications Trust.

[151] Codron D., Lee-Thorp J.A., Sponheimer M., Codron J. (2007). Nutritional Content of Savanna Plant Foods: Implications for Browser/Grazer Models of Ungulate Diversification. Eur. J. Wildl. Res..

[152] Ramachandran V. (2007). Wild Edible Plants of the Anamalais, Coimbatore District, Western Ghats, Tamil Nadu. Indian J. Tradit. Knowl..

[153] Asfaw A., Lulekal E., Bekele T., Debella A., Tessema S., Meresa A., Debebe E. (2023). Ethnobotanical Study of Wild Edible Plants and Implications for Food Security. Trees For. People.

[154] Omotayo A.O., Aremu A.O. (2020). Underutilized African Indigenous Fruit Trees and Food–Nutrition Security: Opportunities, Challenges, and Prospects. Food Energy Secur..

[155] Awodoyin R.O., Olubode O.S., Ogbu J.U., Balogun R.B., Nwawuisi J.U., Orji K.O. (2015). Indigenous fruit trees of tropical Africa: Status, opportunity for development and biodiversity management. Agric. Sci..

[156] Tesfay A., Tewolde-Berhan S., Birhane E., Rannestad M.M., Gebretsadik A., Hailemichael G., Haile M., Gebrekirstos A. (2024). Edible Indigenous Fruit trees and Shrubs in Tigray, Ethiopia. Trees For. People.

[157] Rasmussen L.V., Watkins C., Agrawal A. (2017). Forest Contributions to Livelihoods in Changing Agriculture-Forest Landscapes. For. Policy Econ..

[158] Kamatou G., Vermaak I., Viljoen A. (2011). An Updated Review of Adansonia digitata: A Commercially Important African Tree. S. Afr. J. Bot..

[159] Bvenura C., Sivakumar D. (2017). The role of wild fruits and vegetables in delivering a balanced and healthy diet. Food Res. Int..

[160] Mokganya M.G. (2019). Documentation and Nutritional Evaluation of Some Wild Edible Fruit Plants and Traditional Vegetables of the Vhembe District Municipality, Limpopo Province, South Africa. Doctoral Dissertation.

[161] Nazarudeen A. (2010). Nutritional Composition of Some Lesser-Known Fruits Used by the Ethnic Communities and Local Folks of Kerala. J. Food Sci. Technol..

[162] Aberoumand A., Deokule S. (2009). Studies on Nutritional Values of Some Wild Edible Plants from Iran and India. Pak. J. Nutr..

[163] Musinguzi E., Kikafunda J.K., Kiremire B.T. (2007). Promoting Indigenous Wild Edible Fruits to Complement Roots and Tuber Crops in Alleviating Vitamin A Deficiencies in Uganda. Afr. J. Food Agric. Nutr. Dev..

[164] Tewolde-Berhan S., Remberg S., Wicklund T. (2015). Wild Fruits as a Cheap and Available Source of Micronutrients. Eur. J. Nutr. Food Saf..

[165] Hegazy A.K., Al-Rowaily S.L., Faisal M., Alatar A.A., El-Bana M.I., Assaeed A.M. (2013). Nutritive Value and Antioxidant Activity of Some Edible Wild Fruits in the Middle East. J. Med. Plant Res..

[166] Kucich D.A., Wicht M.M. (2016). South African Indigenous Fruits–Underutilized Resource for Boosting Daily Antioxidant Intake Among Local Indigent Populations?. S. Afr. J. Clin. Nutr..

[167] Akinnifesi F.K., Kwesiga F., Mhango J., Chilanga T., Mkonda A., Kadu C.A.C., Kadzere I., Mithofer D., Saka J.D.K., Sileshi G. (2006). Towards the development of miombo fruit trees as commercial tree crops in southern Africa. For. Trees Livelihoods.

[168] Lekhuleni I.L., Shabalala A., Maluleke M.K. (2024). Quality Aspects of Marula (*Sclerocarya birrea*) Fruit, Nutritional Composition, and the Formation of Value-Added Products for Human Nutrition: A Review. Discov. Food.

[169] Akinnifesi F.K., Leakey R.R., Ajaui O.C., Sileshi G., Tchoundjeu Z., Matakala P., Kwesiga F.R. (2008). Indigenous Fruit Trees in the Tropics: Domestication, Utilization and Commercialization.

[170] Hillman Z., Mizrahi Y., Beit-Yannai E. (2008). Evaluation of Valuable Nutrients in Selected Genotypes of Marula (*Sclerocarya birrea* ssp. caffra). Sci. Hortic..

[171] Moyo M., Kulkarni M.G., Finnie J.F., Van Staden J. (2009). After-Ripening, Light Conditions, and Cold Stratification Influence Germination of Marula [*Sclerocarya birrea* (A. Rich.) Hochst. subsp. caffra (Sond.) Kokwaro] Seeds. HortScience.

[172] Hancock R.D., Viola R. (2005). Improving the Nutritional Value of Crops Through Enhancement of L-Ascorbic Acid (Vitamin C) Content: Rationale and Biotechnological Opportunities. J. Agric. Food Chem..

[173] Hamidou A., Iro D.G., Boubé M., Malick T.S., Ali M. (2014). Potential Germination and Initial Growth of *Sclerocarya birrea* (A. Rich.) Hochst, in Niger. J. Appl. Biosci..

[174] Sybille B., Suarez C., Beckett K. (2012). Marula Fruit: The Next Beverage Innovation. Nutraceutic. Bus. Technol..

[175] Hare Krishna H.K., Saroj P.L., Maheshwari S.K., Singh R.S., Meena R.K., Ram Chandra R.C., Avinash Parashar A.P. (2019). Underutilized Fruits of Arid and Semi-Arid Regions for Nutritional and Livelihood Security. Int. J. Minor Fruits Med. Aromat. Plants.

[176] Ndabikunze B.K., Masambu B.N., Tiisekwa B.M. (2010). Vitamin C and Mineral Contents, Acceptability and Shelf Life of Juice Prepared from Four Indigenous Fruits of the Miombo Woodlands of Tanzania. Int. J. Biol. Chem. Sci..

[177] Shackleton S.E., Shackleton C.M., Cunningham T., Lombard C., Sullivan C.A., Netshiluvhi T.R. (2002). Knowledge on *Sclerocarya birrea* subsp. caffra With Emphasis on its Importance as a Non-Timber Forest Product in South and Southern Africa: A Summary: Part 1: Taxonomy, Ecology and Role in Rural Livelihoods. S. Afr. For. J..

[178] Hall J.B., O’Brien E.M., Sinclair F.L. (2002). Sclerocarya birrea: A Monograph.

[179] Mokgolodi N.C., Ding Y.F., Setshogo M.P., Ma C., Liu Y.J. (2011). The Importance of an Indigenous Tree to Southern African Communities with Specific Relevance to its Domestication and Commercialization: A Case of the Marula Tree. For. Stud. China.

[180] Kunene E.N., Nxumalo K.A., Ngwenya M.P., Masarirambi M.T. (2020). Domesticating and Commercialisation of Indigenous Fruit and Nut Tree Crops for Food Security and Income Generation in the Kingdom of Eswatini. Curr. J. Appl. Sci. Technol..

[181] Mngadi S., Moodley R., Jonnalagadda S.B. (2019). Elemental Composition and Nutritional Value of the Edible Fruits of Transvaal red milkwood (*Mimusops zeyheri*) and Impact of Soil Quality. Environ. Monit. Assess..

[182] Chivandi E., Mukonowenzou N., Nyakudya T., Erlwanger K.H. (2015). Potential of Indigenous Fruit-Bearing Trees to Curb Malnutrition, Improve Household Food Security, Income and Community Health in Sub-Saharan Africa: A Review. Food Res. Int..

[183] Monyela S. (2021). Characterisation of Mmupudu (*Mimusops zeyheri*) Leaf Rust in Limpopo Province.

[184] Mashela P., Mollel N. (2001). Farmer-Identified Indigenous Fruit Tree With Suitable Attributes for the Semi-Arid Northern Province of South Africa. S. Afr. J. Agric. Ext..

[185] Chivandi E., Mukonowenzou N., Berliner D. (2016). The Coastal Red-Milkwood (*Mimusops caffra*) Seed: Proximate, Mineral, Amino Acid and Fatty Acid Composition. S. Afr. J. Bot..

[186] Lockett C.T., Calvert C.C., Grivetti L.E. (2000). Energy and Micronutrient Composition of Dietary and Medicinal Wild Plants Consumed During Drought. Study of Rural Fulani, Northeastern Nigeria. Int. J. Food Sci. Nutr..

[187] Avakoudjo H.G.G., Hounkpèvi A., Idohou R., Koné M.W., Assogbadjo A.E. (2020). Local Knowledge, Uses, and Factors Determining the Use of Strychnos spinosa Organs in Benin (West Africa). Econ. Bot..

[188] Bruschi P., Mancini M., Mattioli E., Morganti M., Signorini M.A. (2014). Traditional Uses of Plants in a Rural Community of Mozambique and Possible Links with Miombo Degradation and Harvesting Sustainability. J. Ethnobiol. Ethnomed..

[189] Aremu A.O., Moyo M. (2022). Health benefits and biological activities of spiny monkey orange (Strychnos spinosa Lam.): An African indigenous fruit tree. J. Ethnopharmacol..

[190] Emmanuel T.V., Njoka J.T., Catherine L.W., Lyaruu H.V. (2011). Nutritive and Anti-Nutritive Qualities of Mostly Preferred Edible Woody plants in Selected Drylands of Iringa District, Tanzania. Pak. J. Nutr..

[191] Raice R.T., Chiau E., Sjoholm I., Bergenstahl B. (2015). The loss of Aroma Components of the Fruit of *Vangueria infausta* L. (*African medlar*) After Convective Drying. Dry. Technol..

[192] Maroyi A. (2018). Nutraceutical and Ethnopharmacological Properties of *Vangueria infausta* subsp. infausta. Molecules.

[193] Steel B., Behr K. (1986). A Small Tree for Rocky Gardens *Vangueria infausta* Wild Medlar. Veld Flora.

[194] Ráice R. (2014). Aroma Components in *Vangueria infausta* L.: Characterization of Components using GC_MS and Aroma Loss During Drying. J. Food Sci..

[195] Ateba C., Kaya H.O., Pitso F.S., Ferim V. (2012). Batswana Indigenous Knowledge of Medicinal and Food Plant Uses for Sustainable Community Livelihood. Afr. Indig. Knowl. Syst. Sustain. Dev. Chall. Prospect..

[196] Moodley R., Koorbanally N., Jonnalagadda S.B. (2013). Elemental Composition and Nutritional Value of the Edible Fruits of *Harpephyllum caffrum* and Impact of Soil Quality on their Chemical Characteristics. J. Environ. Sci. Health Part B.

[197] Low Y.W., Rajaraman S., Tomlin C.M., Ahmad J.A., Ardi W.H., Armstrong K., Athen P., Berhaman A., Bone R.E., Cheek M. (2022). Genomic Insights into Rapid Speciation Within the World’s Largest Tree Genus Syzygium. Nat. Commun..

[198] Semenya S.S., Mokgoebo M.J. (2020). The Utilization and Conservation of Indigenous Wild Plant Resources in the Limpopo Province, South Africa. Nat. Resour. Manag. Biol. Sci..

[199] Işik K. (2011). Rare and Endemic Species: Why are they Prone to Extinction?. Turk. J. Bot..

[200] Moraswi I., Bamigboye S.O., Tshisikhawe M.P. (2019). Conservation Status and Threats to Vascular Plant Species Endemic to Soutpansberg Mountain Range in Limpopo Province, South Africa. Int. J. Plant Biol..

